# The Varieties of the Psychedelic Experience: A Preliminary Study of the Association Between the Reported Subjective Effects and the Binding Affinity Profiles of Substituted Phenethylamines and Tryptamines

**DOI:** 10.3389/fnint.2018.00054

**Published:** 2018-11-08

**Authors:** Federico Zamberlan, Camila Sanz, Rocío Martínez Vivot, Carla Pallavicini, Fire Erowid, Earth Erowid, Enzo Tagliazucchi

**Affiliations:** ^1^Departamento de Física, Universidad de Buenos Aires, Buenos Aires, Argentina; ^2^Instituto de Física de Buenos Aires (IFIBA) and National Scientific and Technical Research Council (CONICET), Buenos Aires, Argentina; ^3^Instituto de Investigaciones Biomédicas (BIOMED) and Technical Research Council (CONICET), Buenos Aires, Argentina; ^4^Fundación Para la Lucha contra las Enfermedades Neurológicas de la Infancia (FLENI), Buenos Aires, Argentina; ^5^Erowid Center, Grass Valley, CA, United States; ^6^UMR7225 Institut du Cerveau et de la Moelle épinière (ICM), Paris, France

**Keywords:** psychedelics, consciousness, phenomenology, binding affinity profile, semantic analysis

## Abstract

Classic psychedelics are substances of paramount cultural and neuroscientific importance. A distinctive feature of psychedelic drugs is the wide range of potential subjective effects they can elicit, known to be deeply influenced by the internal state of the user (“set”) and the surroundings (“setting”). The observation of cross-tolerance and a series of empirical studies in humans and animal models support agonism at the serotonin (5-HT)_2A_ receptor as a common mechanism for the action of psychedelics. The diversity of subjective effects elicited by different compounds has been attributed to the variables of “set” and “setting,” to the binding affinities for other 5-HT receptor subtypes, and to the heterogeneity of transduction pathways initiated by conformational receptor states as they interact with different ligands (“functional selectivity”). Here we investigate the complementary (i.e., not mutually exclusive) possibility that such variety is also related to the binding affinity for a range of neurotransmitters and monoamine transporters including (but not limited to) 5-HT receptors. Building on two independent binding affinity datasets (compared to “*in silico*” estimates) in combination with natural language processing tools applied to a large repository of reports of psychedelic experiences (Erowid’s Experience Vaults), we obtained preliminary evidence supporting that the similarity between the binding affinity profiles of psychoactive substituted phenethylamines and tryptamines is correlated with the semantic similarity of the associated reports. We also showed that the highest correlation was achieved by considering the combined binding affinity for the 5-HT, dopamine (DA), glutamate, muscarinic and opioid receptors and for the Ca+ channel. Applying dimensionality reduction techniques to the reports, we linked the compounds, receptors, transporters and the Ca+ channel to distinct fingerprints of the reported subjective effects. To the extent that the existing binding affinity data is based on a low number of displacement curves that requires further replication, our analysis produced preliminary evidence consistent with the involvement of different binding sites in the reported subjective effects elicited by psychedelics. Beyond the study of this particular class of drugs, we provide a methodological framework to explore the relationship between the binding affinity profiles and the reported subjective effects of other psychoactive compounds.

## Introduction

“*But there are many components of a drug’s action, like the harmonics from the fundamental to the inaudible which, taken in concert, defines the drug. With musical instruments, these components can be shown as sine waves on an oscilloscope. (…) But in psychopharmacology? There is no psychic oscilloscope. There are no easily defined and measured harmonics or phase angles. Certainly, any eventual definition of a drug will require some such dissection into components each of which makes some contribution to the complex whole. The mental process may some day be defined by a particular combination of these components.”*

—(Shulgin and Shulgin, 1995).

Psychedelics are psychoactive substances remarkable for their capacity to elicit a wide range of idiosyncratic effects on consciousness of the self and the environment, as well as changes in perception, emotion and cognition (Nichols, 2016; Carhart-Harris et al., 2018; Preller and Vollenweider, 2018). For millenia, different cultures have adopted the ceremonial use of plants and fungi containing psychedelic molecules. These “classic” psychedelics include mescaline (present in cacti such as peyote, *Lophophora williamsii*), psilocybin (primarily found in the mushrooms of *Psilocybe* genus) and N,N-Dimethyltryptamine (DMT; an orally inactive compound enabled by the combination with β-carbolines in ayahuasca, a brew originally from the Amazon basin; Schultes and Hofmann, 1979; Rätsch, 2005; Nichols, 2016). The discovery of the psychedelic properties of lysergic acid diethylamide (LSD) by A. Hofmann in 1943 provided the first example of a semi-synthetic classic psychedelic, and signaled a period of intense scientific investigation on the subjective effects^1^ elicited by these substances, their mechanism of action in the brain, and their therapeutic potential (Hofmann, 1980).

Experiments both in humans and animal models have provided strong evidence that the psychedelic effects of these molecules are mediated by at least partial agonism at serotonin (5-HT)_2A_ receptors, with a possible role for agonism at other 5-HT receptor subtypes such as 5-HT_2C_ and 5-HT_1A_ (Glennon et al., 1983, 1984; Spencer et al., 1987; Fiorella et al., 1995; Vollenweider et al., 1998; Halberstadt et al., 2011; Hanks and González-Maeso, 2012; Quednow et al., 2012; Kometer et al., 2013; Rickli et al., 2016; Kraehenmann et al., 2017a,b; Preller et al., 2017). These experiments, together with the observation of cross-tolerance between classic psychedelics (Balestrieri and Fontanari, 1959; Isbell et al., 1959, 1961; Appel and Freedman, 1968), led to the consolidation of the serotonergic hypothesis of psychedelic action (Nichols, 2016). This hypothesis states that psychedelics elicit their effects via a common mechanism based on agonism at a relatively small set of 5-HT receptor subtypes. The existence of such mechanism agrees with early studies showing that, in spite of substantial variation in chemical structure, the subjective effects induced by classic psychedelics such as mescaline, psilocybin, DMT and LSD can be considered as similar (Wolbach et al., 1962). Though distinctions have been reported (Coyle et al., 2012; Sanz et al., 2018), they have been attributed to variations in the internal state of the user (“set”) and the surrounding (“setting”; Studerus et al., 2012), as well as dose, which can be difficult to control in non-laboratory settings.

The objective of the present work is to investigate the variety of subjective effects elicited by different psychedelic molecules and to empirically study possible mechanisms underlying such diversity. A large body of anecdotal experiences supports the existence of differences in the subjective effects of serotonergic psychedelics, in particular concerning those elicited by relatively novel synthetic derivatives of phenethylamines (i.e., mescaline analogs) and tryptamines (i.e., DMT analogs). A frequently cited example is that of N,N-Diisopropyltryptamine (DiPT), a substituted tryptamine and 5-HT_1A/2A_ agonist remarkable for producing auditory distortions, in contrast to the predominantly visual effects of classic psychedelics (Shulgin and Carter, 1979; Shulgin and Shulgin, 1997; Kometer and Vollenweider, 2016). Adding to this particular example, over 200 psychoactive substituted phenethylamines and tryptamines presenting an ample range of reported subjective effects are described in the work of A. Shulgin (Shulgin et al., 1961, 1969; Shulgin and Shulgin, 1995, 1997). While most of these compounds are either confirmed or suspected serotonergic psychedelics, others (such as 3,4-methylenedioxyamphetamine [MDA] and 3,4-methylenedioxymethamphetamine [MDMA]) act primarily as monoamine transporter substrates that facilitate the presynaptic release of 5-HT, dopamine (DA) and norepinephrine and have received the alternative denomination of “entactogens” (Nichols, 1986).

The aforementioned evidence for the variety of subjective effects elicited by serotonergic psychedelics presents a challenge to the single-receptor hypothesis of psychedelic action. It has been suggested that such variety could be explained by functional selectivity, i.e., ligand-dependent selectivity for certain intracellular pathways (Urban et al., 2007; Seifert, 2013; Zhou and Bohn, 2014; López-Giménez and González-Maeso, 2018). As stated by D. Nichols: “*Specific agonists with particular substitution patterns may be able selectively to activate a subset of effectors, a phenomenon now known as functional selectivity. It seems likely that functional selectivity can at least partially explain some of the differences reported for the human psychopharmacology of hallucinogens. To date there has been no attempt to correlate specific signaling pathways with any aspect of human psychopharmacology of hallucinogens*” (Nichols, 2017). A complementary possibility (i.e., not mutually exclusive) to that of functional selectivity is that different psychedelic molecules present distinct binding affinity profiles for receptors of neuromodulators and neurotransmitters other than 5-HT (e.g., DA, norepinephrine, histamine, glutamate) and may also act as monoamine transporter substrates.

This work presents a quantitative evaluation of this possibility. We first tested the correlation between the similarity of the reported subjective effects elicited by 18 psychedelic compounds and the similarity of their binding affinity profiles assayed at 42 possible binding sites, as well as the correlation of both with a metric of molecular structure similarity. We also replicated the main result using an independent set of binding affinity measurements at 14 different receptors. In spite of limitations affecting the binding affinity data (see the “Discussion” section), the observation of a significant positive correlation represents preliminary support for the involvement of different receptors in the reported subjective effects. Our work also introduces a novel quantitative and data-driven method based on natural language processing tools to study correlations between pharmacological action and reported subjective experiences.

## Materials and Methods

### Analysis Overview

**Figure 1:**
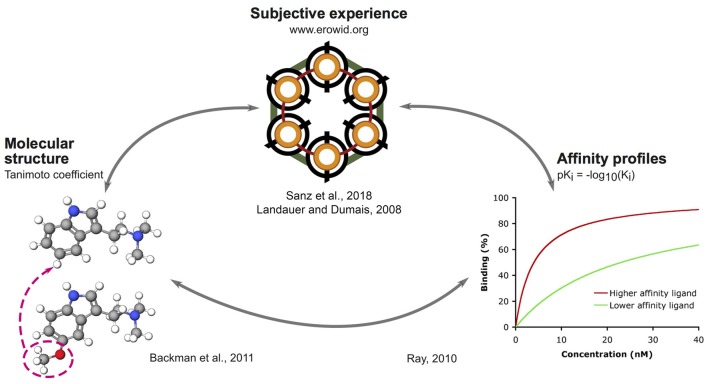
Outline of the methodology followed to link reported subjective effects, binding affinity profiles and molecular structures.

**Figure 2:**
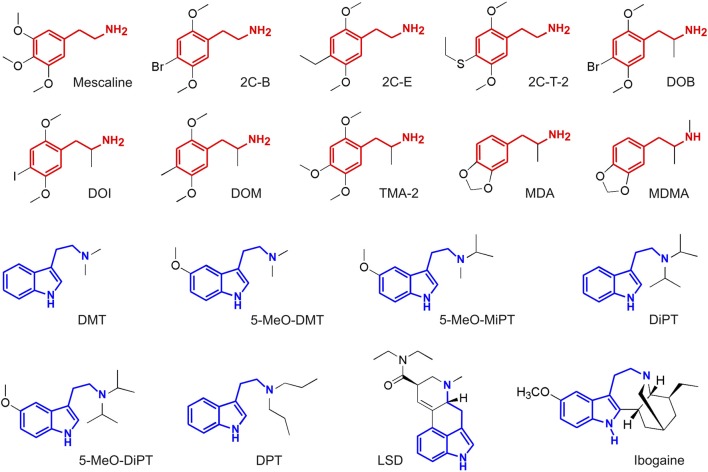
Molecular structure of the compounds selected from Ray (2010), with the phenethylamine and tryptamine functional groups highlighted in red and blue, respectively. Full chemical names and abbreviations are provided in the “Materials and Methods” section.

**Figure 3:**
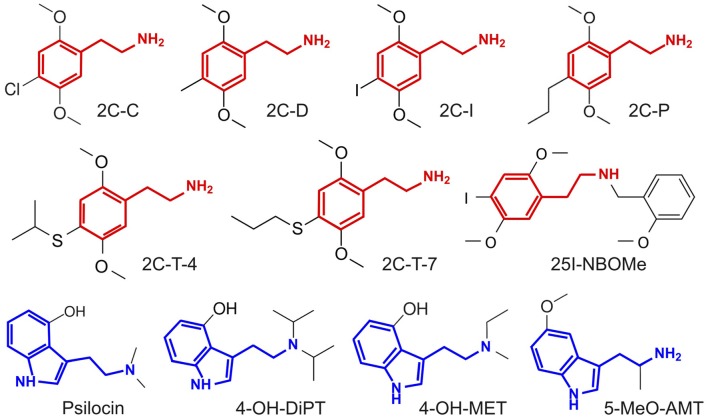
Molecular structure of the compounds selected from Rickli et al. (2015, 2016), with the phenethylamine and tryptamine functional groups highlighted in red and blue, respectively. Full chemical names and abbreviations are provided in the “Materials and Methods” section.

The outline of our analysis is presented in Figure 1. Each node in the diagram represents a data source and the method followed to compute the similarity between different compounds. The psychoactive drugs investigated in this study are shown in Figure 2 (Ray, 2010) and Figure 3 (Rickli et al., 2015, 2016), with the phenethylamine and tryptamine functional groups highlighted in red and blue, respectively. The upper node in Figure 1 represents data on the reported subjective effects of the compounds, which was provided by Erowid Center’s Experience Vaults (Erowid et al., 2017). Latent Semantic Analysis (LSA; Landauer, 2006) was used to compute the semantic similarity between sets of reports of subjective effects corresponding to the different psychedelic compounds, following our analysis in a previously published article (Sanz et al., 2018). The left node in Figure 1 corresponds to data on the molecular structure of compounds included in this study (identified using simplified molecular-input line-entry system [SMILES] strings). The 2D molecular structure similarity was computed using ChemMine Tools^2^ (Backman et al., 2011). Finally, the right node in Figure 1 corresponds to data on the binding affinity profiles of the molecules (Ray, 2010; Rickli et al., 2015, 2016), obtained from assays performed by the NIMH Psychoactive Drug Screening Program (PDSP)^3^ and by researchers from the University of Basel in Switzerland, as described in Rickli et al. (2015, 2016). The similarity between the binding affinity profiles of each pair of compounds was obtained as the Pearson’s linear correlation coefficient of their sign-inverted and log-scaled equilibrium dissociation constants (pK_i_).

### Binding Affinity Similarity

Data on the binding affinity of 18 psychedelic molecules was obtained from tables in the supplementary material of Ray (2010). This data was complemented with binding affinity measurements of 19 compounds published in Rickli et al. (2015, 2016), which were used to provide independent validation of the main results of our analysis.

Two primary sources of data comprise the binding affinities published in Ray (2010). Of the drugs investigated in this work, data for LSD and ibogaine were obtained from the literature. For LSD this information came from NIMH-PDSP assays (Nichols et al., 2002), while the data for ibogaine was compiled from different sources (Ray, 2010). New PDSP binding assays were conducted for the remaining molecules using the methodology described in Ray (2010) and Glennon et al. (2000). Of all sites tested only 42 presented at least one “hit” (i.e., equilibrium dissociation constant K_i_ < 10,000 nm). These sites included 5-HT receptors (5-HT_2A_, 5-HT_2C_, 5-HT_2B_, 5-HT_1A_, 5-HT_1B_, 5-HT_1D_, 5-HT_1E_, 5-HT_5A_, 5-HT_6_, 5-HT_7_), dopamine receptors (D_1_, D_2_, D_3_, D_4_, D_5_), adrenergic receptors (*α*_1A_, *α*_1B_, *α*_2A_, *α*_2B_, *α*_2C_, *β*_1_, *β*_2_), serotonin transporter (SERT), DA transporter (DAT), norepinephrine transporter (NET), imidazoline1 receptor (I_1_), sigma receptors (*σ*_1_, *σ*_2_), delta opioid receptor (DOR), kappa opioid receptor (KOR), mu opioid receptor (MOR), muscarinic receptors (M_1_, M_2_, M_3_, M_4_, M_5_), histamine receptors (H_1_, H_2_), cannabinoid receptors (CB_1_, CB_2_), calcium+ ion channel (Ca+) and N-methyl D-aspartate (NMDA) glutamate receptor. The procedure described in Ray (2010) was followed for the normalization of the *K*_i_ values^4^. A logarithmic transformation with a sign inversion was applied (since higher affinities result in lower *K*_i_ values). This transformation was given by *pK*_i_ = −log_10_(*K*_i_; Neubig et al., 2003). Since the *K*_i_ values generated by the NIMH-PDSP assays were capped at 10,000, the lowest possible *pK*_i_ value was −4 (note that *pK*_i_ = −4 was also assigned in other circumstances, as described in Ray (2010); e.g., when the primary assay did not produce >50% inhibition). All the *K*_i_ values are available as online Supplementary Material in Ray (2010); as well as in Supplementary Table S1 (raw *K*_i_ values) and Supplementary Table S2 (normalized *pK*_i_ values) of the Supplementary Material.

The radioligand binding assays conducted in Rickli et al. (2015, 2016) are described with detail elsewhere (Hysek et al., 2012; Simmler et al., 2013). Briefly, measurements were performed at membrane preparations of human embryonic kidney cells. Affinity values (Ki) were derived from IC_50_ values (Cheng-Prusoff equation) obtained from 3–5 independently replicated concentration-response curves; this procedure was performed at the following binding sites: 5-HT receptors (5-HT_1A_, 5-HT_2A_, 5-HT_2C_), dopamine receptors (D_1_, D_2_, D_3_), adrenergic receptors (*α*_1A_, *α*_2A_), the histamine H_1_ receptor, trace amine-associated receptors (TAAR_1_, from rat and mouse genes), SERT, DAT and NET. A logarithmic transformation was applied as described above to obtain the *pK*_i_ values. The supplementary information includes the analysis (Figure A1) of activation potency measurements (EC_50_) at 5-HT_2A_ receptors and of monoamine transporter inhibition at NET, DAT and SERT. Details of these experiments are provided in Rickli et al. (2015, 2016).

Finally, to compare the binding affinities of two psychedelic compounds, we computed the Pearson’s linear correlation coefficient between the corresponding sets of *pK*_i_ values. When applied to all pairs of compounds, this resulted in matrices containing all correlation coefficients. Whenever the compounds under comparison presented missing data at a subset of sites, the correlation coefficient was computed using only the *pK*_i_ values that were simultaneously available for both compounds.

### Selection of Psychedelic Compounds

A subset of the molecules investigated in Ray (2010) was included in the present study (Figure 2). These partially overlap with those investigated in Rickli et al. (2015, 2016); those that were assayed in the latter but not in the former are presented in Figure 3. We followed two criteria for inclusion of compounds in our study. First, we discarded molecules lacking binding affinity data at most of the sites. Second, only compounds with a sufficiently high (>10) number of subjective reports in the Erowid database were included. The application of these criteria to the data presented in Ray (2010) resulted in the inclusion of 18 compounds, 10 of which were classified as substituted phenethylamines and seven as substituted tryptamines. The same criteria applied to the data provided in Rickli et al. (2015, 2016) resulted in the inclusion of 19 compounds (11 phenethylamines and eight tryptamines). LSD belongs to the class of ergolines, which can be considered as a subtype of tryptamines (“rigidified tryptamines,” Nichols, 2016). Therefore, for reasons of simplicity, we classified LSD as a tryptamine (note however that the phenethylamine functional group can be found in the tetracyclic ring structure of ergolines). For the purposes of this work, this classification is analogous to that into phenylalkylamines (which includes phenethylamines) and indolealkylamines (which include tryptamines, but also ergolines and β-carbolines; Glennon, 1994).

The 18 compounds from Ray (2010) that were included in this study are the following:

- Mescaline (3,4,5-Trimethoxyphenethylamine): naturally occurring substituted phenethylamine, first identified in 1897 by A. Heffter and synthesized in 1919 by Spath (1919).
- 2C-B (4-Bromo-2,5-dimethoxyphenethylamine): substituted phenethylamine, first synthesized in 1974 by A. Shulgin (Shulgin and Shulgin, 1995). Missing data for β_1_, β_2_ and Ca+.
- 2C-E (4-Ethyl-2,5-dimethoxyphenethylamine): substituted phenethylamine, first synthesized in 1977 by A. Shulgin (Shulgin and Shulgin, 1995). Missing data for β_2_, σ_1_,
- 2C-T-2 (4-Ethylthio-2,5-dimethoxyphenethylamine): substituted phenethylamine, first synthesized in 1981 by A. Shulgin (Shulgin and Shulgin, 1995).
- DOB ([±]-2,5-Dimethoxy-4-bromoamphetamine): substituted phenethylamine, first synthesized in 1967 by A. Shulgin (Shulgin and Shulgin, 1995).
- DOI ([±]-2,5-Dimethoxy-4-iodoamphetamine): substituted phenethylamine, first synthesized in 1973 by Coutts and Malicky (1973). Missing data for Ca+.
- DOM ([±]-2,5-Dimethoxy-4-methylamphetamine): substituted phenethylamine, first synthesized in 1963 by A. Shulgin (Shulgin and Shulgin, 1995). Missing data for M_3_, M_4_ and Ca+.
- TMA-2 ([±]-2,4,5-Trimethoxamphetamine): substituted phenethylamine, first synthesized in 1933 by V. Bruckner and assayed in 1962 by A. Shulgin (Shulgin and Shulgin, 1995). Missing data for I_1_, σ_1,_ σ_2_, DOR, KOR, MOR, M_1_, M_3_, M_4_, CB_1_, CB_2_ and NMDA.
- MDA ([±]-3,4-Methylenedioxyamphetamine): substituted entactogen phenethylamine, first synthesized in 1910 by G. Mannish and W. Jacobson and tested by G. Ailes in 1930 (Mannich and Jacobsohn, 1910). Missing data for σ_1,_ σ_2_, DOR, MOR, M_1_, M_3_, M_4_, H_1_, Ca+ and NMDA.
- MDMA ([±]-3,4-Methylenedioxymethamphetamine): substituted entactogen phenethylamine, first synthesized by A. Köllisch at MERCK laboratories in 1912 and not explored in humans until the 1970s (McDowell and Kleber, 1994). Missing data for σ_1_, σ_2_, DOR and CB_2_.
- DMT (N,N-Dimethyltryptamine): naturally occurring substituted tryptamine that is ubiquitous within the kingdom Plantae, as well as endogenous to the human brain (Barker et al., 1981). It was first synthesized in 1931 by Manske (1931). Missing data for DOR, MOR, H_1_ and NMDA.
- 5-MeO-DMT (5-Methoxy-N,N-dimethyltryptamine): naturally occurring substituted tryptamine present in a variety of plant species and a toad species (Shulgin and Shulgin, 1995). It was first synthesized in 1936 by Hoshino and Shimodaira (1936). Missing data for DOR, MOR, H_1_ and NMDA.
- 5-MeO-MiPT (5-Methoxy-N-methyl-N-isopropyltryptamine): substituted tryptamine, first synthesized by Repke et al. (1985). Missing data for CB_1_.
- DiPT (N,N-Diisopropyltryptamine): substituted tryptamine, first synthesized and tested by Shulgin and Carter (1979).
- 5-MeO-DiPT (5-Methoxy-N,N-diisopropyltryptamine): substituted tryptamine, first synthesized and tested by Shulgin and Carter (1979).
- DPT (N,N-Dipropyltryptamine): substituted tryptamine, first reported in 1971.
- LSD (lysergic acid diethylamide): synthetic ergoline first produced by A. Hofmann in 1938 and subsequently tested by himself in 1943 (Hofmann, 1980). Missing data for α_2B_, α_2C_, I_1_, σ_1_, σ_2_, M_1_, M_2_, M_3_, M_4_, M_5_, H_2_, CB_1_, CB_2_, Ca+ and NMDA.

Ibogaine, a naturally occurring tryptamine which can be found in plants of the Apocynaceae family (Schultes and Hofmann, 1979). The synthesis of ibogaine was achieved in 1956 by M. M. Janot and R. Goutarel (Goutarel and Janot, 1956). Missing data for 5-HT_2B_, 5-HT_1E_, 5-HT_5A_, 5-HT_6_, 5-HT_7_, D_4_, D_5_, α_1A_, α_1B_, α_2A_, α_2B_, α_2C_, β_2_, NET, I_1_, M_4_, M_5_, H_2_, CB_1_, CB_2_ and Ca+. While ibogaine is used primarily in ritual and therapeutic contexts, the range of receptors involved (including, but not limited to, the 5-HT_2A_ receptor; Sweetnam et al., 1995) and its equally heterogenous subjective effects allow for variability that is of advantage when investigating the correlation between both dimensions.

The additional compounds assayed in Rickli et al. (2015, 2016) and included in this study are the following:

- 2C-C (4-Chloro-2,5-dimethoxyphenethylamine): substituted phenethylamine, first synthesized in 1984 by Cheng and Castagnoli (1984).
- 2C-I (2,5-Dimethoxy-4-iodophenethylamine): substituted phenethylamine, first synthesized and tested in 1976 by A. Shulgin (Shulgin and Shulgin, 1995).
- 2C-P (2,5-Dimethoxy-4-propylphenethylamine): substituted phenethylamine, first synthesized and tested by A. Shulgin (Shulgin and Shulgin, 1995).
- 2C-T-4 (2,5-Dimethoxy-4-isopropylthiophenethylamine): substituted phenethylamine, first synthesized and tested by A. Shulgin (Shulgin and Shulgin, 1995).
- 2C-T-7 (2,5-Dimethoxy-4-propylthiophenethylamine): substituted phenethylamine, first synthesized and tested by A. Shulgin (Shulgin and Shulgin, 1995).
- 2C-D (2,5-Dimethoxy-4-methylphenethylamine): substituted phenethylamine, first synthesized and tested by Ho et al. (1970).
- 25I-NBOMe (2,5-Dimethoxy-4-iodo-N-(2-methoxybenzyl)phenethylamine): substituted phenethylamine, first synthesized by Heim (2004).
- 4-OH-MET (4-Hydroxy-N-methyl-N-ethyltryptamine): substituted tryptamine and close analog of psilocin, first synthesized by A. Shulgin (Shulgin and Shulgin, 1997).
- 5-MeO-AMT: substituted alpha-methylated tryptamine, first synthesized by A. Shulgin and D. Nichols (Shulgin and Shulgin, 1997).
- 4-OH-DiPT (4-Hydroxy-diisopropyltryptamine): substituted tryptamine, first synthesized by Repke et al. (1977) and investigated in humans by A. Shulgin (Shulgin and Shulgin, 1997).
- Psilocin (4-Hydroxy-N,N-dimethyltryptamine): naturally occurring substituted tryptamine, primarily found in mushrooms of the *Psilocybe* genus and metabolized *in vivo* from psilocybin.

While many reports of experiences with mushrooms of the *Psilocybe* genus (containing psilocybin as their main active compound) were available, we decided not to use them as a proxy for the isolated active principle. Mushrooms can contain other psychoactive alkaloids (e.g., baeocystin and norbaeocystin) capable of influencing the subjective effects (Leung and Paul, 1968). They may also contain phenethylamine, which could underlie some of the undesired effects associated with consumption of *Psilocybe* mushrooms, such as tachycardia, nausea, anxiety (Beck et al., 1998). A survey reported that these undesired effects were frequently observed in users who consumed mushrooms, but not in those who consumed isolated psilocybin (van Amsterdam et al., 2011). Finally, interoception (understood as the sensing of peripheral bodily signals) is known to play a role in anticipatory events that can modulate behavior and subjective effects even before a drug has time to reach the brain and elicit its pharmacological action (Wise et al., 2008). Hofmann and Wasson speculated that the chewing of mushrooms could elicit a faster onset of the subjective effects relative to ingesting a pill containing psilocybin, because in the former case psilocybin can be absorbed by the mucous membrane, while in the latter case the pills must first reach the stomach and dissolve before psilocybin can enter the system (an account of this speculation in the context of a traditional mushroom ceremony carried out with pills containing psiloybin instead of mushrooms can be found in Hofmann, 1980).

### Semantic Similarity of Subjective Effects Reports

Reports of the subjective effects of the psychedelic compounds included in this study were acquired from Erowid’s Experience Vaults^5^. Erowid Center is a nonprofit, member-supported organization providing access to reliable, non-judgmental information about psychoactive plants, chemicals and related issues, containing a large number (>20,000) of published reports of the subjective effects of different psychoactive substances. The full Erowid corpus and the classification of its reports into different categories is extensively described in a previous work (Sanz et al., 2018). A total of 16 reports were obtained for mescaline, 143 for 2C-B, 206 for 2C-E, 101 for 2C-T-2, 36 for DOB, 32 for DOI, 23 for DOM, 19 for TMA-2, 63 for MDA, 770 for MDMA, 236 for DMT, 247 for 5-MeO-DMT, 69 for 5-MeO-MiPT, 45 for DiPT, 182 for 5-MeO-DiPT, 137 for DPT, 718 for LSD, 32 for ibogaine, 64 for 2C-C, 383 for 2C-I, 57 for 2C-P, 16 for 2C-T-4, 171 for 2C-T-7, 48 for 2C-D, 144 for 25I-NBOMe, 51 for 4-OH-MET, 109 for 5-MeO-AMT, 208 for 4-OH-DiPT and 8 for psilocin/psilocybin. All the Erowid users authorize the use of their narratives with research purposes at the time of submission. The data obtained from Erowid’s Experience Vaults was completely anonymous, consisting of lists of terms derived from the tokenization of all the reports associated with each substance, without links to individual reports nor the users who submitted them. The measures followed to secure data confidentiality and privacy at the Erowid Center servers are described on its website. This data is both accessible online and available upon request at the Erowid Center for its use with research purposes.

The preprocessing of the reports was performed using the Natural Language Toolkit (NLTK^6^) in Python 3.4.6 (Bird, 2006). The reports were first separated into individual words after discarding all punctuation marks. Each word was lemmatized using NLTK (i.e., converted to the root from which it is inflected). All words were converted to lowercase and lemmatized words containing less than three characters were discarded. As described in a previous work (Sanz et al., 2018), words including substance names, slang variations and terms related to possible routes of administration were removed from the reports.

To quantify the semantic similarity between the reports of the psychedelic compounds included in this study we applied LSA (Landauer, 2006), a natural language processing technique based on the hypothesis that words with similar meaning appear with similar frequency in texts (Sahlgren, 2008). The rationale behind LSA is comparing the profile of word frequencies after reducing the rank of the word-by-document matrix, thus attenuating the problems of sparseness and lack of context-sensitive term occurrence. Before applying LSA we computed the frequency of the different words using the term frequency-inverse document frequency (tf-idf) transform, as implemented in scikit-learn^7^ (Pedregosa et al., 2011; Leskovec et al., 2014). The tf-idf transform computes a matrix in which rows are unique words in the corpus and columns represent “documents” (for this analysis, a “document” contains all the reports associated with a psychoactive compound contained in the published corpus from the Erowid Experience Vaults, see Sanz et al., 2018). The product of the term frequency and the inverse document frequency determines the entries of this matrix. The term frequency is defined as the number of times each term appears in each document. The inverse document frequency is defined as the logarithmically scaled inverse fraction of the documents containing the term. To eliminate very frequent/rare terms from the corpus, only those terms appearing in more/less than 5%/95% of the documents were retained, resulting in a vocabulary of 1,466 words.

The word-by-document matrix obtained using the tf-idf transform was decomposed into the product of three matrices using Singular Value Decomposition (SDV; Klema and Laub, 1980). Of the three resulting matrices (U, S, V), S contains in its diagonal the matrix of singular values ordered by size, and U and V are real unitary matrices (their size is determined by the number of words and documents, respectively). To reduce the number of linearly independent rows (terms) while preserving the similarity structure among columns (documents), only the first D largest singular values were retained and all others were set to zero, resulting in the reduced matrix of singular values S*. Following previous work (Sanz et al., 2018), we retained the *D* = 20 largest singular values. Computing the product of the matrices U, S*, V yields a low-rank approximation of the word-by-document frequency matrix.

Finally, the correlation matrix containing the Pearson’s linear correlation coefficient between the columns of the rank-reduced word-by-document matrix (corresponding to each of the drugs) was computed as a metric of the semantic similarity of the reports of each pair of compounds.

### Structural Similarity

To measure the 2D structural similarity between the molecular structures of the compounds we computed atom pair similarities using the Tanimoto coefficient, as implemented in ChemMine Tools^8^ (Backman et al., 2011). Each molecule was represented as a set of atom pairs, comprising pairs of non-hydrogen atom types together with the distances between them (computed as the lengths of the shortest bond-by-bond paths between them, Sheridan et al., 1996). The Tanimoto coefficient provides an index ranging between 0 and 1, obtained as the size of the intersection of the atom pair sets divided by the size of the union of the atom pair sets. This method has been shown to perform more efficiently than other metrics and is a standard tool to quantify 2D molecular similarity (Chen and Reynolds, 2002; Willett, 2006).

### Principal Components of Subjective Reports

We applied principal component analysis (PCA) to reduce the word-by-document matrix into a smaller number of components that capture idiosyncratic features of the reported experiences in the Erowid database. PCA was performed using an algorithm based on SVD as implemented in MATLAB 2014 (Wall et al., 2003). We retained the first five components explaining the highest variance in the tf-idf rank-reduced word frequency matrices. For each component we obtained 1,466 coefficients, which were used to represent in word cloud format,^9^ the words that were most representative of each component.

The scores of each document (i.e., the combined set of subjective reports of a given psychoactive substance in the Erowid corpus) were used to produce radar plots quantifying the presence of the five principal components in the narratives. By correlating the binding affinity of each receptor with the corresponding principal component scores across the 18 compounds, we produced radar plots quantifying the relevance of each receptor/transporter/Ca+ channel for each of the five principal components.

### Virtual Screening of Binding Affinity Profiles

Following the methodology introduced by Vidal and Mestres (2010) suggesting that an unknown interaction of a ligand for a particular target can be approximated by its molecular resemblance to a selection of assayed active compounds, we implemented a similar “*in silico*” approach for the virtual screening of binding affinity profiles (Vidal and Mestres, 2010). The main objective of this analysis was to obtain an independent set of binding affinity values to compare with those reported in Ray (2010) and evaluate the plausibility given the relatively low number of replicates.

Using the open-source data mining and integration platform KNIME^10^ (Berthold et al., 2009), we extracted the molecular structures with their respective assayed binding affinity values from two public access chemical databases: ChEMBL^11^ (Gaulton et al., 2012) curated by the European Bioinformatics Institute (EBI) of the European Molecular Biology Laboratory (EMBL), and the KiDatabase^12^ (Evans et al., 2001) from the PDSP of the National Institute of Mental Health (NIMH). The molecular descriptor summarizing the structure of each compound as a binary (“bit”) vector was calculated using the Fingerprint node of the open-source cheminformatics extension RDKit for KNIME^13^. The similarity between the fingerprints of a pair of molecules was obtained using the Tanimoto distance coefficient (described in a previous section). For this purpose, the Fingerprint Similarity node from the KNIME-CDK Integration was used (Beisken et al., 2013). With the objective of minimizing the noise due to the contribution of compounds without a sufficiently high structural correlation with the drugs included in this study, we implemented the filtration criteria proposed by Paulke et al. (2013) consisting of a cut-off similarity value equivalent to the 95% quantile of the overall similarity distribution. Finally, the *K*_i_ values were predicted by inverse distance weighting, assigning different contribution weights to each molecule in the databases that are inversely proportional to the structural difference between the molecules:

$${\text{K}}_{i}^{\text{j}}(\text{m})=\frac{\sum_{\text{i}}{\text{w}}_{i}(\text{m}){\text{u}}_{i}}{\sum_{\text{i}}{\text{w}}_{i}(\text{m})};\;{\text{w}}_{i}(\text{m})=\frac{1}{\text{d}{\left(\text{m},{\text{x}}_{i}\right)}^{\text{p}}}$$

where ${\text{K}}_{i}^{\text{j}}(\text{m})$ represents the binding affinity value of the molecule m (i.e., one of those presented in Figure 2) at the j-th receptor, u_i_ represents the binding affinity of the i-th ligand at the databases included in the analysis, and w_i_(m) is a weighting term for the affinity value based on an exponent of the inverse distance between the molecule m and the x_i_ ligand. The distance was computed as one minus the Tanimoto coefficient based on different molecular descriptor algorithms. The best power parameter value “p” and the most suitable molecular descriptor algorithm were obtained after running an iterative optimization procedure benchmarked against the known binding affinity profile of 13 non-selective psychiatric drugs assayed by Roth et al. (2004). Using the optimal value *p* = 4.0 and the “Layered” algorithm gave a 39% of hits against 34 receptors, an acceptable result compared to the reference value of 59.3%–66.7% achieved by Vidal and Mestres (2010) using the same procedure applied to a larger database of compounds.

## Results

### Correlation Between the Similarity of Reported Subjective Effects, Binding Affinity Profiles and Molecular Structures

**Figure 4:**
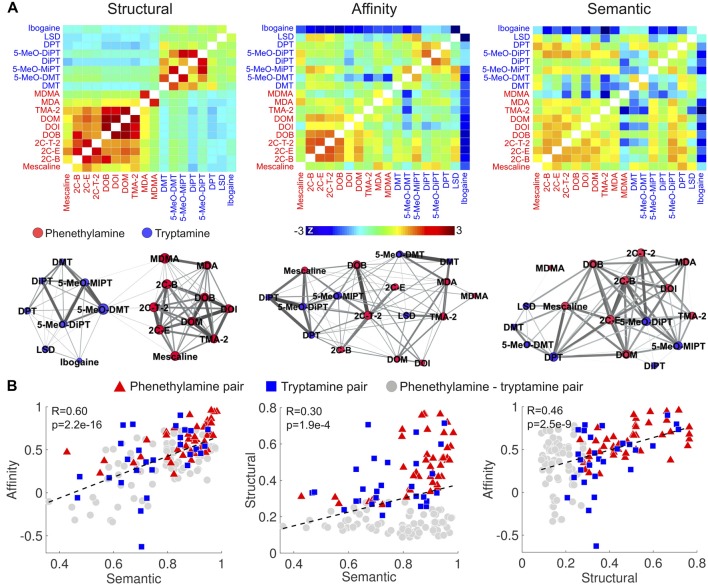
Correlation between the similarities of reported subjective effects, binding affinity profiles and molecular structures. **(A)** Top: matrices containing the pairwise structural, binding affinity profile and subjective effects similarities of the 18 compounds. To uniformize the color scales, we converted all entries to z-scores by subtracting the means and dividing by the variances. Bottom: graph representation of the pairwise similarities. Each node corresponds to compound, node sizes represent the sum of the similarities to all other compounds, and edge sizes represent the similarities between the linked nodes. **(B)** Scatter plots of the binding affinity profile similarity vs. subjective effects similarity (left), molecular structure similarity vs. subjective effects similarity (center) and binding affinity profile similarity vs. molecular structure similarity (right). Dashed lines represent the best linear fit in the least squares sense. Pearson’s linear correlation coefficients (*R*) and the associated *p*-values (*p*) are shown in the insets.

We first computed the similarities between the compounds in terms of the reported subjective effects, binding affinity profiles and molecular structures based on the data provided in Ray (2010). These are presented in matrix and graph forms in Figure 4A (graphs were visualized using Gephi^14^ with the “force atlas” layout; Bastian et al., 2009). It is clear from the matrix and graph representations of the molecular similarity that the Tanimoto coefficient differentiates phenethylamines from tryptamines. 2C-x and DOx compounds presented high within-group structural similarity, as well as MDA and MDMA. As expected, tryptamines and their methoxylated analogs (e.g., DMT and 5-MeO-DMT) presented a high degree of structural similarity. Ibogaine and LSD presented a moderate similarity to other tryptamines (likely a consequence of their complex multi-cyclic structures) and a lower similarity to phenethylamines. Such clear differentiation between tryptamines and phenethylamines was not as marked for the similarity of binding affinity profiles and the reported subjective effects. Compounds of the 2C-x family, tryptamines and their methoxylated analogs presented the highest similarities in terms of binding affinity profiles, while ibogaine presented a generally low similarity to the other compounds (however, this could be influenced by the relatively high amount of missing data for ibogaine—see the “Materials and Methods” section).

As shown in Figure 4B, we observed positive and significant correlations between the binding affinity profile similarity and the reported subjective effects similarity (*R* = 0.60, *p* = 2.2e–16), between the binding affinity profile similarity and the molecular structure similarity (*R* = 0.46, *p* = 2.5e–9), and between the reported subjective effects similarity and molecular structure similarity (*R* = 0.3, *p* = 1.9e–4). To test whether these correlations were driven by two clusters presenting high/low within/between group similarities, we employed different symbols for phenethylamine-phenethylamine, tryptamine-tryptamine and phenethylamine-tryptamine pairs in the scatter plots of Figure 4B. These clusters are not apparent in the visualization of the data. We observed that correlations restricted to phenethylamine-tryptamine pairs were in all cases significant (*p* < 1e–4).

**Figure 5:**
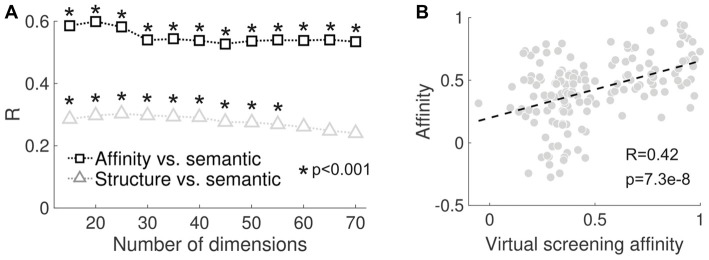
**(A)** Correlation between reported subjective effects similarity and molecular structure similarity (black) and between reported subjective effects similarity and binding affinity profile similarity (gray) plotted as a function of the number of dimensions retained after performing SVD in the latent semantic analysis (LSA) algorithm. **(B)** Scatter plot of the binding affinity profile similarity computed using the values provided in Ray (2010) and those obtained by the implemented virtual screening procedure. The dashed line represents the best linear fit in the least squares sense. The Pearson’s linear correlation coefficient (*R*) and the associated *p*-value (*p*) are shown in the inset.

We tested how the correlation values and their significance depended on the number of components retained in the SVD step of LSA (see “Materials and Methods” section). As shown in Figure 5A, both sets of correlation coefficients (reported subjective effects vs. molecular structure/binding affinity profiles) remained positive, but declined as a function of the number of components retained. We also employed a previously validated computational framework for the “*in silico*” estimation of binding affinity profiles (“virtual receptorome screening”). This approach consisted of querying large databases for ligands that have been experimentally assayed at certain sites and that are also structurally close to the molecules in Figure 2. The binding affinities were then estimated by the average of the experimental data of all those ligands weighted by the structural similarity with each of the psychedelic molecules included in the present study (see the “Virtual Screening of Binding Affinity Profiles” section of the “Materials and Methods” for more information). This method was implemented to provide an independent evaluation of the feasibility of the PDSP data provided in Ray (2010); as well as of the data compiled from different sources. The significant positive correlation (*R* = 0.42, *p* = 7.3e–8) between the empirical and estimated binding affinity profiles supports such feasibility (see Figure 5B).

### Replication of the Correlation Between the Similarity of Reported Subjective Effects and the Binding Affinity Profiles

**Figure 6:**
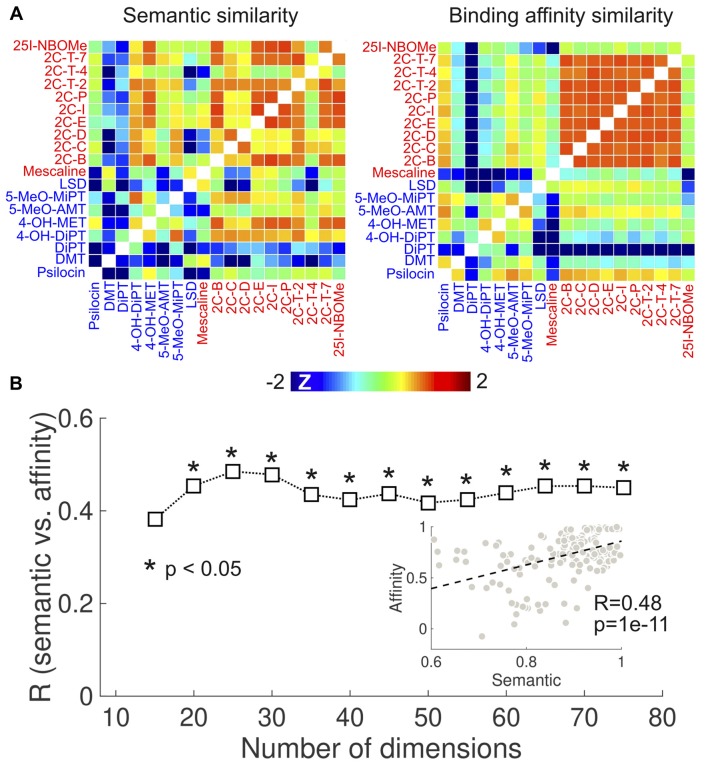
Correlation between the similarities of reported subjective effects and the binding affinity profiles, based on data from Rickli et al. (2015, 2016). **(A)** Matrices containing the pairwise binding affinity profile (right) and reported subjective effects similarities (left) of the 19 compounds selected from Rickli et al. (2015, 2016). To uniformize the color scales, we converted all entries to z-scores by subtracting the means and dividing by the variances. **(B)** Correlation between reported subjective effects similarity and binding affinity profile similarity as a function of the number of dimensions retained in the LSA algorithm. The scatter plot in the inset represents the binding affinity profile similarity vs. subjective effects similarity (for 25 dimensions), including the associated Pearson’s linear correlation coefficient *(R)* and the corresponding *p*-value *(p)*. The dashed line indicates the best linear fit in the least squares sense.

To provide independent support for the results in Figure 4, we investigated the correlation between the semantic similarity of the reported subjective effects and the similarity of the binding affinity profiles using the *K*_i_ values published in Rickli et al. (2015, 2016). The results of this analysis are presented in Figure 6. Figure 6A shows the semantic (retaining 20 dimensions in the LSA algorithm) and binding affinity similarity matrices. We observed high binding affinity profile similarities for 2C-x compounds, consistent with the results obtained from the PDSP data (Figure 4). 25I-NBOMe presented a relatively lower similarity to other phenethylamines. Also consistent with Figure 4, tryptamines presented lower within-group similarity in terms of binding affinities and of the reported subjective effects. Conversely, phenethylamines presented higher within-group similarity in both senses. Figure 6B shows the correlation coefficient between the semantic and binding affinity profile similarities as a function of the number of dimensions. We observed *R* > 0.4 except for the lowest number of dimensions. The scatter plot in the inset provides an example of the correlation between both variables (*R* = 0.48, *p* = 1e–11).

### Optimization of Receptor Selection for the Prediction of the Reported Subjective Effects and Molecular Structure Similarities

The correlations reported in Figure 4B correspond to those obtained using the binding affinities measured at all 42 sites (Ray, 2010). It could be the case that higher correlations are achievable when considering only a subset of those sites. This would imply that the binding affinities at those sites are of a higher relative importance to explain the subjective effects of the compounds. We first evaluated whether the binding affinity profiles restricted to 5-HT receptor subtypes yielded a higher correlation than the reported in Figure 4B for all 42 sites, which did not occur (*R* = 0.31). This suggested the relevance of other neurotransmitters and neuromodulators for the prediction of the similarity of the reported subjective effects.

**Figure 7:**
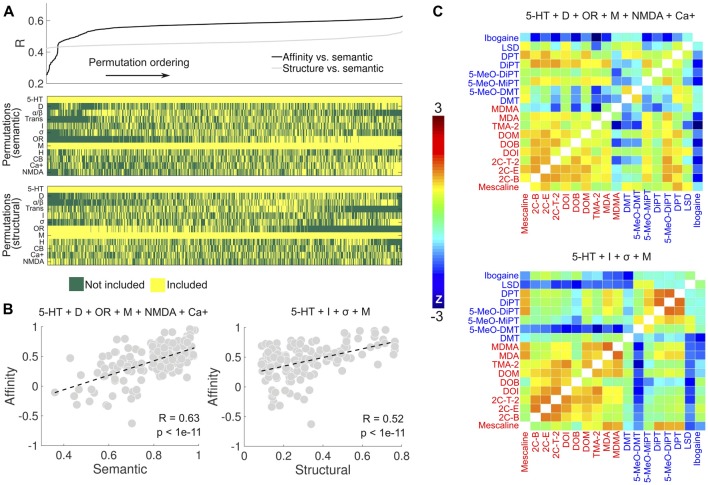
Optimization of receptor selection for the prediction of the subjective effects and molecular structure similarities. **(A)** Top: linear correlation coefficients (sorted, ascending) between binding affinity profile and subjective effects similarities (black), and between binding affinity profile and molecular structure similarities (gray). The binding affinity profile similarities were computed using all combinations of receptor types, transporter proteins and the Ca+ channel (5-HT, serotonin receptors; D, dopamine receptors; *α*/*β*, adrenergic receptors, Trans.; serotonin/dopamine/norepinephrine transporters, I, Imidazoline1 receptor; *σ*, sigma receptors; OR, delta, kappa and mu opioid receptors; M, muscarinic receptors; H, histamine receptors; CBs, cannabinoid receptors; Ca+, calcium+ channel; NMDA, N-methyl D-aspartate glutamate receptor), with the restriction that at least one of 5-HT, D, *α*/*β* and M had to be included. Bottom: the combinations corresponding to each linear correlation value shown in the panel above. **(B)** Left: scatter plot of the binding affinity profile similarity vs. subjective effect similarity for the set of binding sites maximizing the linear correlation coefficient (5-HT + D + OR + M + NMDA + Ca+). Right: scatter plot of the binding affinity profile similarity vs. molecular structure similarity for the set of receptors maximizing the linear correlation coefficient (5-HT + I + *σ* + M). Dashed lines represent the best linear fit in the least squares sense. Pearson’s linear correlation coefficients *(R)* and their associated *p*-values *(p)* are shown in the insets. **(C)** Top: matrix containing the pairwise binding affinity profile similarity, computed using the set of binding sites optimizing the prediction of subjective effects similarity. Bottom: matrix containing the pairwise binding affinity profile similarity, computed using the binding sites optimizing the prediction of molecular structure similarity.

We explored the combinations of receptor types to find the one that maximized the correlations in Figure 4B. The search space was restricted to combinations including at least one of the following groups of receptors: 5-HT, dopamine (D), adrenergic (*α*/*β*) and muscarinic (M). This restriction ensured that sufficient data points were available for the computation of the correlation coefficients. Figure 7A shows the sorted linear correlation coefficients between the binding affinity profile similarity and reported subjective effects similarity (black), and between the binding affinity profile similarity and the molecular structure similarity (gray). The panels below display the combinations of receptors/transporters/Ca+ channel corresponding to each correlation value shown above. The optimal combination of sites (*R* = 0.63, *p* < 1e–17) to predict the reported subjective effects similarity from the binding affinity profile similarity was based on data from 5-HT, DA, opioid, muscarinic, NMDA receptors and the Ca+ channel (Figure 7B, left). The optimal prediction (*R* = 0.52, *p* < 1e–11) of the molecular structure similarity was based on the binding affinity profile restricted to 5-HT, imidazoline, σ and muscarinic receptors (Figure 7B, right). In Figure 7C we show the matrices containing the pairwise binding affinity profile similarity, restricted to the set of receptors obtained by the optimization procedures described above. The resemblance with the reported subjective effects and molecular structure similarity matrices shown in Figure 4A is apparent upon direct visual inspection.

**Figure 8:**
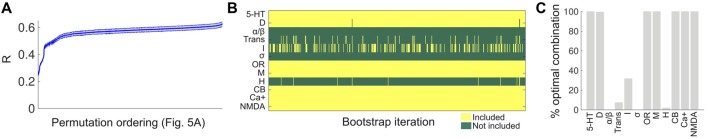
Bootstrap analysis of the receptor selection optimization. **(A)** The average linear correlation coefficient between binding affinity profile similarity and subjective effects similarity across 1,000 bootstrap iterations. For each iteration, the correlation coefficients were computed for all the combinations shown in Figure 7A, and plotted in the same ordering (mean ± STD). **(B)** The columns of the matrix contain the optimal selection of receptors/transporters/Ca+ channel for each bootstrap iteration. **(C)** The percentage of times a receptor type/transporter/Ca+ channel appeared in set that maximized the correlation between the binding affinity profile similarity and the subjective effects similarity.

To assess the robustness of the results presented in Figure 7, we conducted a bootstrap analysis with 1,000 iterations, applying in each iteration the LSA algorithm to 50% of the words in the Erowid reports (randomly chosen, with replacement). Each iteration yielded a correlation coefficient between the binding affinity profile similarity and the reported subjective effects similarity, for each combination of receptors/transporters/Ca+ channel shown in Figure 7A. The average correlation coefficients across all 1,000 bootstrap iterations are shown in Figure 8A, using the same ordering of the permutations as in Figure 7A. Even though small fluctuations occurred, a similar functional dependence was observed, implying that the combinations explored in Figure 7A produced very similar correlation coefficients across all bootstrap iterations. Figure 8B shows the optimal selection of receptors/transporters/Ca+ channel for all bootstrap iterations in matrix form. It is clear that certain receptors were frequently included (e.g., 5-HT receptors) while others were systematically excluded. Figure 8C shows the percentage of times (over all 1,000 iterations) that a receptor type/transporter protein/Ca+ channel appeared in the set that maximized the correlation between the binding affinity profile similarity and the reported subjective effects similarity. 5-HT, DA, opioid, muscarinic, cannabinoid and NMDA receptors and the Ca+ channel were selected for all or almost all bootstrap iterations.

### Principal Components of the Reports of Subjective Effects

**Figure 9:**
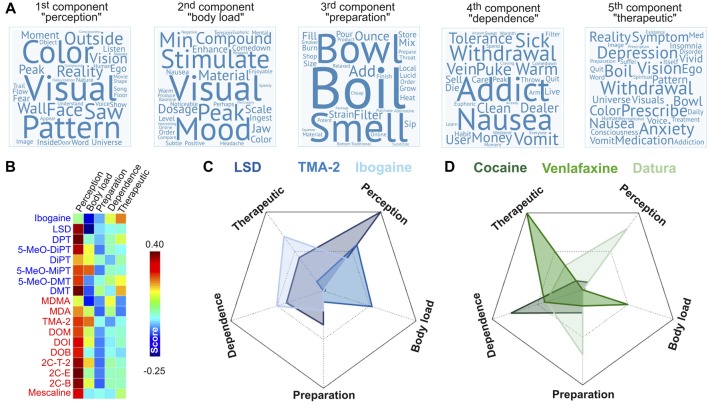
Principal components of the subjective effects reports. **(A)** Word clouds representing the most relevant terms in the first five principal components of the Erowid corpus (Sanz et al., 2018), ordered as a decreasing function of explained variance. **(B)** Scores of the principal components for each psychedelic compound. **(C)** Radar plot showing the principal component scores of 3 of the 18 psychedelic compounds. **(D)** Radar plot showing the principal component scores of three psychoactive compounds in the Erowid database that were not investigated in this study. Radar plots are scaled so that the minimum (possibly negative) value of each axis is located at the vertex.

The next step of our analysis was to identify the facets of the reported subjective experiences linked to the degree of binding affinity at the 42 sites published in Ray (2010). For this purpose, we applied PCA to the word-by-document rank-reduced matrix obtained after applying LSA to the tf-idf matrix (see “Materials and Methods” section). This analysis included all the reports of psychoactive substances in the Erowid corpus as described in a previous publication (Sanz et al., 2018). We retained the first five principal components, which explained ≈58% of the variance of the data. The coefficients associated to each word indicated its relevance for each component and were used as the weights to construct the word clouds presented in Figure 9A. The five principal components are summarized below:

- 1st component (“perception”), variance explained ≈23%. Top 10 ranking words: visuals, color, visual, pattern, saw, reality, face, their, outside, vision.
- 2nd component (“body load”), variance explained ≈13%. Top 10 ranking words: visuals, stimulation, mood, compound, peak, material, visual, dosage, minute, comedown.
- 3rd component (“preparation”), variance explained ≈9%. Top 10 ranking words: boil, bowl, smell, add, ounce, filter, strain, pour, material, mix.
- 4th component (“dependence”), variance explained ≈7%. Top 10 ranking words: addict, addiction, withdrawal, nausea, vomit, sick, money, puke, warm, tolerance.
- 5th component (“therapeutic”), variance explained ≈5%. Top 10 ranking words: withdrawal, depression, anxiety, prescribe, vision, symptom, nausea, boil, medication, reality.

The names of the principal components were chosen to indicate the nature of their most relevant terms. The “perception” component presented high weights for terms associated with conscious perception of the self (e.g., “ego,” “fear”) and the environment (e.g., “visual,” “color”). While the “body load” component also included such terms, others associated with stress and negative bodily sensations also presented high weights (e.g., “nausea,” “tension,” “headache,” “jaw,” “stimulate”). The “preparation” component was clearly associated with the processing and preparation of psychoactive materials. The remaining two components (“dependence” and “therapeutic”) were labeled following similar considerations. Words relevant for the “dependence” component included terms such as “addict,” “addiction,” “withdrawal,” and “tolerance,” as well as others that could be related to the adverse reactions observed during the withdrawal syndrome of some addictive drugs (“nausea,” “vomit,” “sick,” “puke”). With respect to the last component (“therapeutic”), many terms of high relevance were directly associated with medical disorders (“depression,” “insomnia,” “disorder,” “anxiety,” “treatment,” “prescribe,” “med,” “symptom”). Words in common with the “dependence” component could represent adverse secondary reactions of medicaments. The inclusion of terms associated with psychedelic or visionary experiences (“universe,” “visuals,” “vision,” “ego”) might indicate the therapeutic use of some of the compounds included in our analysis.

Figure 9B shows the score of each principal component for the 18 psychedelic compounds included from Ray (2010). As expected, the “perception” component presented the highest score for all compounds (except ibogaine), with the highest scores corresponding to 2C-E, DPT and DMT. In terms of the “body load” component, the highest scores were observed for 5-MeO-MIPT, TMA-2 and 2C-T-2; in terms of the “preparation” component were DMT, DiPT and 5-MeO-DMT; in terms of the “dependence” component were MDMA, ibogaine and 5-MeO-DMT and in terms of the “therapeutic” component were the same three as in the “dependence” component. Figure 9C presents radar plots showing the principal component scores of three compounds included in this study (LSD, TMA-2 and ibogaine), while Figure 9D presents an analogous plot for three psychoactive compounds included in the Erowid database but not analyzed in this study (cocaine, venlafaxine and plants of the Datura genus; Sanz et al., 2018). These substances presented scores agreeing with their known subjective effects, “set” and “setting”; e.g., cocaine scored high in the “dependence” component, venlafaxine in the “therapeutic” component, and plants of the Datura genus in the “perception” and “preparation” components.

### Correlations Between Receptor Binding Affinities and Principal Component Scores

**Figure 10:**
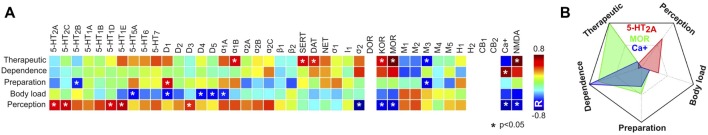
Correlations between receptor binding affinities and principal component scores. **(A)** Matrix containing the Pearson’s linear correlation coefficients between principal component scores (rows) and binding affinities (columns) at the 42 sites, computed across 18 psychedelic compounds (Ray, 2010). Blank spaces indicate that it was not possible to compute the linear correlation value due to missing data. **(B)** Radar plot showing the correlations in **(A)** for three binding sites with different profiles (5-HT_2A_ binding affinity presented the highest correlation with the “perception” component scores, mu opioid receptor (MOR) with the “therapeutic” and “dependence” component scores, and Ca+ with the “dependence” component scores).

Knowing the principal component scores for each psychedelic compound, as well as their receptor binding affinities, it was possible to obtain their correlation and thus to determine whether a higher binding affinity for a certain receptor predicted higher principal component scores and vice-versa. The results of this analysis are presented in Figure 10A. The most salient results were the positive correlations between binding affinities at some 5-HT receptor subtypes and the score of the “perception” component. We observed generally negative correlations for the “body load” component, and positive correlations between affinity at transporters (SERT, DAT), opioid (MOR, KOR) and NMDA receptors and the “therapeutic” component scores, while Ca+ affinity correlated positively with the “dependence” component scores. Figure 10B shows a radar plot for the correlation at sites presenting different profiles (5-HT_2A_, MOR, Ca+).

## Discussion

We have performed a novel synthesis of data on the reported subjective effects of 18 psychedelic compounds, their affinities at 42 possible binding sites and their molecular structure (Ray, 2010). We supported this analysis with a replication using an independent dataset of 19 compounds assayed at 14 sites (Rickli et al., 2015, 2016). While it is acknowledged that certain psychedelic molecules act at non-5-HT receptors (Nichols, 2016) and that binding assays suggest affinity for an ample range of sites (Ray, 2010; Rickli et al., 2015, 2016), quantitative analyses of reported subjective effects are required to evaluate potential polypharmacological influences on the consciousness-altering properties of psychedelics. Performing controlled experiments for this evaluation is severely limited by the obstacle of administering scheduled drugs to participants and assessing their subjective experiences in a laboratory setting (Nutt et al., 2013). We were able to elude this limitation by the use of self-reported narratives of the subjective effects elicited by psychedelic compounds (Erowid et al., 2017). In the following, we present a discussion of our findings and how they relate to current theories about the mechanisms behind psychedelic effects in the human brain, and what factors might underlie the variability of these effects. Finally, we discuss the limitations of our study, with an emphasis on those related to the binding affinity data.

Our findings include evidence supporting a variety of reported subjective effects in compounds of similar molecular structure, as seen in the semantic similarity matrix shown in Figure 4A, as well as in Figure 5. While the distinction between entactogens such as MDMA and MDA and serotonergic psychedelics is well-established both in terms of neuropharmacology and phenomenology (Nichols, 1986), there is scarcer evidence supporting such distinction for the other substituted tryptamines and phenethylamines analyzed in this study (with the possible exception of ibogaine, known to produce effects that only partially overlap with those of classic psychedelics, Sweetnam et al., 1995; Schenberg et al., 2017). Early human studies reported a failure to successfully discriminate between the acute effects of classic psychedelics such as LSD, psilocybin and mescaline (Hollister and Hartman, 1962; Wolbach et al., 1962). The use of animal models trained in drug discrimination tasks provided more mixed results (Winter, 2009; Baker, 2017), even though it has been suggested that drug discrimination capacity in animals is not related to the typical subjective effects experienced by humans (Baker, 2017). Some of the most salient distinctions between classic psychedelics are related to their pharmacokinetics. While some of these differences can be explained by metabolic processes outside the central nervous system, recent research shows that the formation of particular ligand-receptor complexes influences the pharmacokinetics of LSD, as well as the activation of specific signaling pathways, which in turn might impact on the potency and subjective effects of the drug (Wacker et al., 2017). Thus, we speculate that differences in terms of pharmacokinetics between classic psychedelics could reflect properties of the receptor-ligand complex and that those properties might also nuance the subjective effects of the drugs. In contrast to classic psychedelics, the phenomenology of more novel synthetic compounds (e.g., 2C-x and DOx families of substituted phenethylamines) is less researched, but anecdotal reports compiled in two volumes by Ann and Alexander Shulgin (Shulgin and Shulgin, 1995, 1997) suggest a variety of subjective effects. Previous analyses of Erowid’s large corpora of experience narratives is consistent with such variety (Coyle et al., 2012; Sanz et al., 2018).

While it is now clear that 5-HT_2A_ agonism underlies the psychedelic effect of certain substituted tryptamines and phenethylamines, the series of events that occur after the molecule interacts with the receptor is likely to depend on the conformation of the ligand-receptor complex (functional selectivity; Urban et al., 2007). In other words, knowing the affinity of a certain ligand for the 5-HT_2A_ receptor still entails uncertainty about cellular responses, and how the differential activation of intracellular signaling events affects the subjective experience elicited by the drug. Functional selectivity has been demonstrated for a class of receptors known as G protein coupled receptors (GPCRs; Zhou and Bohn, 2014). While the canonical 5-HT_2A_ signaling pathway depends on Gαq coupling and activation of phospholipase C (PLC; Conn and Sanders-Bush, 1986), evidence exists showing that the canonical activation of PLC does not suffice to explain the mechanism of action of psychedelics (Sanders-Bush et al., 1988; Egan et al., 1998; Rabin et al., 2002), and that other complex signaling mechanisms (such as that mediated by phospholipase A_2_ [PLA_2_]) are differentially activated by 5-HT_2A_ psychoactive ligands, which in turn induce specific patterns of intracellular signaling and behavioral responses (Moya et al., 2007).

Elucidating the chain of events leading from differential signaling pathway activation to the subjective effects elicited by different psychedelic molecules is currently beyond our capabilities (Nichols, 2016, 2017). However, a future project that systematically characterizes the activation profiles of PLC, PLA_2_, β-arrestins and other intracellular signaling events for different psychedelic compounds could yield valuable data for the correlation with the associated reported subjective effects. Such data could contribute towards establishing indirect links between the profiles of the activated intracellular signaling events and the qualitative aspects of the associated psychedelic experiences. However, our findings suggest that functional selectivity at the 5-HT_2A_ receptor (and possibly at other 5-HT receptor subtypes) may not be the sole contributor to the variety of reported subjective effects. As shown in Figure 4, the similarity of the reported subjective effects can be at least partially predicted by the binding affinity profiles at a wide range of receptors and transporter proteins. This result was replicated in an independent dataset, as shown in Figure 5. Such positive correlations would not be expected if the nature of the elicited subjective effects depended solely on functional selectivity at the 5-HT_2A_ receptor. It must be noted, however, that both mechanisms are not mutually exclusive and are likely to act in combination. In particular, it is clear that (partial) agonism at the 5-HT_2A_ receptor is a necessary condition to enable the effects of psychedelic compounds. The situation becomes more complicated after considering that some of the other neurotransmitters investigated in this study (e.g., DA, histamine, norepinephrine, glutamate, acetylcholine, endorphins and cannabinoids) also bind to GPCR (Urban et al., 2007; Zhou and Bohn, 2014), and that each of them could influence the subjective effects via functional selectivity. Even though it is clear that a large-scale project is required to establish an explanatory bridge between the action of psychedelics at the molecular level and their consciousness-altering properties, we believe that our methodological approach suggests the direction towards the first steps in this direction.

Even when considering the possibility that affinity for an ample range of receptors mediates psychedelic action, the reverse process of tuning the set of binding sites to achieve an optimal prediction of the reported subjective effects similarities revealed the redundancy of certain binding sites (i.e., histaminic, σ, *α*/*β* receptors and monoamine transporters) However, the sole consideration of 5-HT receptor subtypes yielded a relatively poor prediction. In particular, our analyses highlighted the relevance of DA receptors (D_1–5_). The dopaminergic action of LSD is well-documented (Nichols, 1976; Watts et al., 1995). It has been proposed that LSD binds first to 5-HT_2A_ receptors, leading to increased sensitivity of the dopaminergic system (primarily the D_2_ and D_4_ receptors; Seeman et al., 2005; Marona-Lewicka et al., 2009) resulting in a biphasic temporal effect (Freedman, 1984; Marona-Lewicka and Nichols, 2007). Previous studies also established that the interaction with DA receptors is relevant for the action of other psychedelics; for instance, it has been reported that the D_2_ receptor inverse agonist haloperidol impaired the subjective effects of psilocybin in humans (Vollenweider et al., 1998), even though silent antagonism of haloperidol at the 5HT_2A_ receptor most likely contributed to the impairment of these effects.

Experimental evidence supports the relevance of group II (mGluR2/3) and NMDA glutamate receptors for the action of psychedelic tryptamines, including the interaction between 5-HT receptor-mediated behavior and glutamate systems (Winter et al., 2004; González-Maeso et al., 2007, 2008; Delille et al., 2012; Carbonaro et al., 2015). It has been speculated that the glutamatergic action of psychedelics could underlie the similarities with the phenomenology and neurophysiology of certain NMDA antagonist arylcyclohexylamines (e.g., ketamine, phencyclidine (PCP); Bowdle et al., 1998; Muthukumaraswamy et al., 2015; Schartner et al., 2017). Our analyses also suggest that affinities for muscarinic and opioid receptors are relevant for the prediction of the reported subjective effects. This is a largely unexplored research area; however, there is at least one example of a compound with high specificity for an opioid receptor presenting strong altering-consciousness properties (salvinorin A, a highly selective and potent KOR agonist; Roth et al., 2002).

The best prediction of molecular structure similarity was achieved considering the binding affinities at 5-HT, imidazoline, muscarinic and *σ* receptors. This is consistent with the differential selectivity for 5-HT receptors between tryptamines and phenethylamines. The latter display higher selectivity for 5-HT_2A_ and 5-HT_2C_ receptors, while the behavioral and subjective effects of tryptamines are also likely to be influenced by 5-HT_1A_ agonism (McKenna et al., 1990; Blair et al., 2000; Nichols, 2016). It also known that DMT regulates *σ* receptors, which may be a property shared with other substituted tryptamines as opposed to substituted phenethylamines (Fontanilla et al., 2009), even though the physiological relevance of this action at *σ* receptors *in vivo* remains unknown. The role of muscarinic and imidazoline receptors in the structural distinction between tryptamines and phenethylamines remains unclear and should be addressed in future studies.

The use of published narratives instead of well-validated psychometric tests (Dittrich, 1998; Studerus et al., 2010) lacks numerical ratings of different items that characterize the psychedelic experience. We applied PCA to the subjective reports and found components that could be identified with effects on consciousness, perception and bodily sensations. However, other components transcended these elementary facets of subjective experience and could be identified with substance dependence, therapeutic use and with the preparation of psychoactive materials. These components contained valuable information, allowing the identification of certain receptors and monoamine transporter proteins with reports of therapeutic use; however, the PCA analysis did not compensate the lack of information provided by psychometric tests. Future efforts include the development of a “mixed” online platform for the submission of free narratives and a structured assessment of the experiences by means of rating a series of numerical items. In spite of these limitations, the PCA analysis yielded results consistent with the known subjective and somatic effects of different drugs. For instance, LSD presented a high score for the “perception” component in contrast to TMA-2, which was reported to induce a higher body load (Shulgin, 1964), and ibogaine presented high scores for the “dependence” and “therapeutic” components, most likely reflecting its use in the treatment of addictions (Popik et al., 1995; Schenberg et al., 2014). An interesting methodological development is the link between the principal components and the binding sites. These results reproduced some known facts about the functional role of different receptors; for instance, the results shown in Figure 10 reflect the known association between the 5-HT_2A_ receptor and the perception-altering properties of psychedelics in humans (Glennon et al., 1983; Spencer et al., 1987; Fiorella et al., 1995; Vollenweider et al., 1998; Halberstadt et al., 2011; Hanks and González-Maeso, 2012; Quednow et al., 2012; Kometer et al., 2013; Rickli et al., 2016; Kraehenmann et al., 2017a,b; Preller et al., 2017).

At this point of the discussion it is instructive to return to the quotation heading the introductory section of this article (Shulgin and Shulgin, 1995). While the concept of decomposing the subjective effects of a drug as the addition of elementary components is theoretically attractive, the analogy with Fourier analysis (i.e., the decomposition into harmonics) is limited in the sense that the Fourier decomposition is linear (i.e., the signal is represented as a weighted sum of different terms). On the other hand, the brain is a highly non-linear system and neuromodulation is not an exception (Chialvo, 2010; Breakspear, 2017). An illustrative example comes from the pharmacology of atypical antipsychotics. While it is well-established that D_2_ receptor antagonism alleviates the positive symptoms of schizophrenia, the brain-wide antagonism of such receptors leads to a number of undesired side effects (Kapur et al., 2000). However, incorporating 5-HT_2A_ antagonism results in a series of complex effects including the reduction of 5-HT_2A_-mediated DA activation and the release of DA that competes with the drug’s D_2_ antagonist effects, reducing antagonistic action in brain areas involved in the side effects (Markowitz et al., 1999). As a result of these interactions between different neuromodulatory systems, atypical antipsychotics perform well despite having lower D_2_ occupancy than first generation drugs. The complex and interrelated nature of neuromodulatory systems in the brain also questions the validity of employing specific antagonists to investigate the role of the antagonized receptors in the reported subjective effects of the drug or in animal behavior. While this experimental paradigm has been fundamental to establish the key relevance of 5-HT_2A_ receptors for psychedelic action, it is uncertain whether it can be extended to manipulate more subtle aspects of the effects elicited by psychedelic drugs (Winter, 2009).

Our study is based on the synthesis of data from diverse sources and therefore it inherits limitations intrinsic to each source of information. As discussed in a recent article (Sanz et al., 2018), the reports in the Erowid database could be contaminated by expectation effects, and most lack laboratory verification of the identity and dosage of the consumed substances, which may be elusive even to the users themselves. Additional potential confounds include lack of information on the history of drug use (i.e., whether users were drug naïve when they consumed the substances, and whether metabolites of other drugs were present, which could affect the nature of the reported experiences), and intrinsic variability in genetic and metabolic traits of the users. However, the large number of reports available from Erowid’s published database could allow a meaningful signal to emerge in spite of these uncontrolled sources of noise (Halevy et al., 2009). The acquisition of such amount of data for many different psychedelic compounds (most of which are currently placed in the Schedule 1 category) in a double-blind placebo-controlled study presents difficulties that are most likely insurmountable within the current legislation (Nutt et al., 2013).

Particular emphasis must be given to the limitations inherent to the binding affinity data. Such values do not inform of the pharmacological action at the GPCRs (i.e., whether the drugs are agonists, partial agonists, antagonists, etc.). Thus, in principle the binding affinity at a given receptor for two molecules included in this study could represent the efficiency to elicit opposite responses. However, lack of functional assay experiments for these compounds may not result in critical confounds under the assumption that very similar (in the structural sense) molecules are unlikely to present opposite actions at the same target. A previous study found that two structurally similar ligands generally occupy the same region in the binding sites at the receptor, citing this observation as support for the use of shape matching in drug design (Boström et al., 2006). This matching between structurally similar ligands may indicate a similar pharmacological action at the receptor. Another limitation of the data employed in our study relates to the influence of the chosen radioligands, which can affect the estimated binding affinity values, especially when comparing measurements based on antagonist vs. agonist radioligands (which could label different populations of the active/inactive conformations of the receptor; Bylund and Toews, 1993).

We emphasize that binding affinity values may not be directly proportional to drug potency (i.e., EC_50_ and IC_50_ values). Such relationship is expected from a simple model of drug-receptor interaction based on the law of mass action; according to this model the potency of a drug is directly proportional to the equilibrium dissociation constant (Kenakin, 2004). In this model, a key assumption is that the intrinsic activity (or efficacy; Stephenson, 1956) of the drug at the receptor is similar to that of a full agonist. We have included in the Appendix an analysis based on data included in Rickli et al. (2015, 2016) and Ray (2010) showing that such approximate relationship is observed across different sets of serotonergic psychedelics at the 5-HT_2A_, 5-HT_2C_ receptors and at the monoamine transporters NET, DAT and SERT. It is uncertain whether this simple model can be applied to all drug-receptor pairs here investigated. However, there is evidence that such relationship holds for 5-HT, DA and monoamine transporters, for which a significant correlation exists between the binding affinity similarities (i.e., affinities restricted to these sites only) and the reported subjective effects similarities (*R* = 0.55, *p* = 9.3e–14; see Figure 7). We also note that dissociation constants reflect chemical/thermodynamical properties of the ligand-receptor interactions, whereas potency measurements are relative, e.g., to the choice of measured response. It is also not clear that *in vitro* measurements of EC_50_/IC_50_ values are more accurate than binding affinities for the purpose of our analyses, given that the *in vivo* (in this case, in humans) elicited response will depend upon the concentration of the drug and its metabolites. Such concentration does not only depend on the consumed dose, but also on metabolism and diffusion through different tissues and membranes. For these reasons, the screening of binding affinities is considered as a reasonable first approximation for the expected *in vivo* activity of a drug (Roth et al., 2004), given the presence of several unknowns that can only be dissipated by obtaining dose-response curves using different response variables (see for instance Drews, 2000: “*The set of binding affinities for a given compound is termed its affinity fingerprint. The similarity of affinity fingerprints has been shown to correlate with the biological activities of druglike substances*”).

Given these limitations, we note that our methodological developments yielded sensible results in spite of unaccounted potential sources of noise in the data. We also note that positive and significant correlations between reported subjective effects and binding affinity profile similarities were obtained from two independent sources of data (Ray, 2010; Rickli et al., 2015, 2016). Future new experiments should be carried out that contribute towards overcoming these limitations, i.e., functional assays, experiments using homogeneous radioligands, the measurement of possible allosteric effects, dose-response curves at other receptors, and a more complete screening in order to avoid missing binding affinity data. The use of “*in silico*” computational modeling may contribute towards overcoming some of these limitations and lend support to experimental data. Our relatively straightforward computational approach to estimate binding affinity profiles presented a significant correlation with the empirical values published in Ray (2010), exemplifying the potential relevance of this type of approach for future studies.

From all possible perspectives, the scientific study of psychedelic drugs is a very young field of research. Unlike other drugs, human studies are unavoidable to fully understand the neuropsychopharmacology of psychedelics (Shulgin and Shulgin, 1995). The most salient feature of these compounds is their capacity to modify the conscious state of the user; thus, their investigation cannot rely solely on animal models lacking the capacity to produce complex reports of conscious content. Unfortunately, sources of reliable data are still scarce, both in terms of the elicited subjective effects and pharmacological measurements *in vivo*. We cannot avoid concluding that our study and its results should be considered as preliminary. Further replications of the binding affinity data measurements are required for a consensus to emerge, thus paving the way towards the application of the proposed methodology to yield more robust and reliable results. We must point out that, while preliminary studies are unavoidable in any young field of research, the future possibilities of conducting replication studies could be hindered by spreading conclusions that are unguaranteed by the obtained results which should, in turn, be backed up by standard and consensual reference data. For these reasons, we have opted to incorporate to our analysis all currently available sources of binding affinity data in order to lend as much support as possible to our relatively limited conclusions, while at the same time developing tools that could become increasingly relevant as the field matures and more reliable sources of data become available. Also, for these reasons, a large portion of our discussion relates to actual or potential limitations of the data, how they could impact the present analyses, and how they could be overcome in future studies.

In conclusion, we proposed and applied a novel method towards the characterization of the reported subjective effects of psychedelic compounds and their relationship to pharmacological data. Future studies may build upon our methodology by linking the semantic similarity of subjective reports to the brain-wide effects of psychedelic compounds as measured using functional neuroimaging techniques (Dos Santos et al., 2016). Our methodology is especially useful for the study of psychedelics, given the aforementioned difficulties inherent to assessing their subjective effects in a laboratory setting. However, we conceive the creation and mining of similar large databases of experiences associated with the use neuropsychiatric medications (e.g., antidepressants and antipsychotics). The combination of this information with chemical, neuropharmacological, neuroimaging and transcriptomics data could allow a “reverse-engineering” procedure (e.g., Figure 7) for the optimization of binding affinity profiles relative to the beneficial and detrimental effects of the drugs.

## Author Contributions

FZ and CS analyzed data. RMV and CP provided critical comments and helped draft an early version of the manuscript. FE and EE contributed materials and towards the interpretation of the Erowid Experience Vault data. ET conceived the analysis, analyzed data, prepared the figures and wrote the final version of the manuscript.

## Conflict of Interest Statement

The authors declare that the research was conducted in the absence of any commercial or financial relationships that could be construed as a potential conflict of interest.

## Figures and Tables

**A1:**
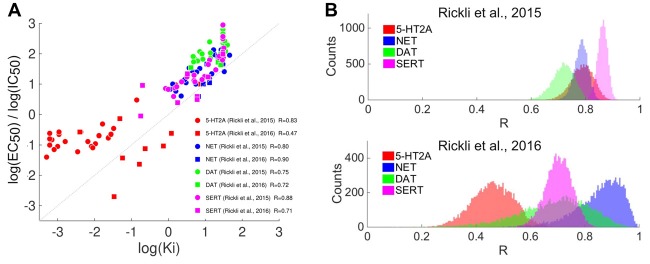
**(A)** Scatter plot of the log(EC_50_) and log(IC_50_) vs. log(*K*_i_) values published in Rickli et al. (2015, 2016). The inset includes the linear correlation coefficients between each set of measurements. **(B)** Estimated distributions of the correlation values between log(EC_50_) and log(IC_50_), and log(*K*_i_), randomly sampled (10.000 iterations) assuming normal distribution of the values, and using the confidence intervals provided in Rickli et al. (2015; upper panel) and Rickli et al. (2016; lower panel).

## Footnotes

^1^Here “subjective effects” stands for drug-induced reportable experiences, which might reflect changes in sensory perception, but also bodily sensations and altered thought processes and cognition.

^2^http://chemmine.ucr.edu/

^3^https://pdspdb.unc.edu/pdspWeb/

^4^We note, however, that our results were preserved using different normalization procedures and, in particular, when working directly with the raw *K*_i_ values. This is shown in Supplementary Figure S1. Furthermore, since the analyses are based on linear correlations, the results are mathematically invariant under multiplicative factors (e.g., choice of measurement units).

^5^https://erowid.org/experiences/

^6^http://www.nltk.org/

^7^www.scikit-learn.org

^8^http://chemmine.ucr.edu/

^9^https://wordart.com/

^10^https://www.knime.com/

^11^https://www.ebi.ac.uk/chembl/

^12^https://pdsp.unc.edu/databases/kidb.php

^13^http://www.rdkit.org/

^14^https://gephi.org/

## Appendix

### Relationship Between Binding Affinity and Potency

As discussed in a paragraph concerning the limitations of the pharmacological data, binding affinities do not necessarily predict EC_50_ and IC_50_ values, as determined from dose-response curves, given that the drugs can present different intrinsic activities (or efficacies) at each of the binding sites (Stephenson, 1956). We determined the correlation between pK_i_ values and −log(EC_50_) values for the 5-HT_2A_ receptor, and the correlation between *pK*_i_ values and −log(IC_50_) values for monoamine transporters (NET, DAT, SERT) for the compounds assayed in Rickli et al. (2015, 2016).

The results are presented in Figure A1. In **(A)**, we show a scatter plot of log(EC_50_) and log(IC_50_) vs. log(*K*_i_) values. It is clear from these figure that both sets of measurements are in approximate direct proportion. The inset of this panel includes the Pearson’s linear correlation coefficients for the compounds assayed in Rickli et al. (2015, 2016) at 5-HT_2A_, NET, DAT and SERT. In **(B)**, we randomly sampled log(EC_50_), log(IC_50_) and log(*K*_i_) values (for which a normal distribution can be assumed) based on the confidence intervals provided in Rickli et al. (2015, 2016)^15^. We then computed the correlation coefficient between the 10,000 randomly sampled log(EC_50_)/log(IC_50_) and log(*K*_i_) values. These distributions indicate an overall positive correlation between binding affinity and drug potency.

Finally, based on activity measurements at 5-HT_2A_ and 5-HT_2C_ receptors reported in Ray (2010), we demonstrated a high correlation between the binding affinities of the NIMH-PDSP assayed compounds and their log(EC_50_) values (*R* = 0.90 for 5-HT_2A_ and *R* = 0.86 for 5-HT_2C_).
