# Supplementary material for: The Varieties of the Psychedelic Experience: A Preliminary Study of the Association Between the Reported Subjective Effects and the Binding Affinity Profiles of Substituted Phenethylamines and Tryptamines
Source: Front Integr Neurosci. 2018 Nov 8;12:54. doi: 10.3389/fnint.2018.00054 (PMC6235949; doi:10.3389/fnint.2018.00054)
Supplement: Supplementary file 1 [file Data_Sheet_1.PDF]

Supplementary tables containing raw and normalized Ki values

Binding site	DOB	MDA	MDMA	Mescaline	2C-B	DMT	5-MeO-DMT	2C-E	2C-T-2	5-MeO-MIPT	DIPT	5-MeO-DIPT	DPT	DOI	DOM	TMA-2	LSD	Ibogaine
5-HT$_{1A}$	2550	3762	>10E4	1841	240	>1E4	1.9	307	381.2	12.3	120	132.4	31.8	2219	3656	>10E4	7.3	>1E4
5-HT$_{1B}$	940	>1E4	>1E4	>1E4	104	>1E4	74	252	857.5	302.8	>1E4	5137	854.4	>1E4	>1E4	>1E4	3.9	>1E4
5-HT$_{1D}$	635	>1E4	>1E4	>1E4	26.3	93	6.3	73.2	85.9	22.5	3742	1718	618.8	457.8	208.9	>1E4	7.8	>1E4
5-HT$_{1E}$	1427	>1E4	>1E4	5205	119	455	360	626	415.1	3496	>1E4	>1E4	2338	1013	3542	>1E4	92.9	NA
5-HT$_{2A}$	23.1	>1E4	>1E4	>1E4	27.6	2323	2011	43.9	39.9	448.3	>1E4	>1E4	2579	165.4	507.4	584.2	11.3	>1E4
5-HT$_{2B}$	3.9	91	500	792	13.5	107	3884	25.1	6	58.5	398.5	163	42	335.9	11.7	154.4	30	NA
5-HT$_{2C}$	41.5	6418	>1E4	>1E4	89.5	334	538	104	53.9	2186	>1E4	>1E4	1567	45.8	3980	4062	30.6	>1E4
5-HT$_{5A}$	5311	>1E4	>1E4	>1E4	>1E4	611	276	>1E4	>1E4	952.9	>1E4	>1E4	4373	>1E4	>1E4	>1E4	9	NA
5-HT$_6$	5535	>1E4	>1E4	>1E4	319	487	35.5	2971	1362	130.2	>1E4	>1E4	4543	2113	8155	>1E4	6.9	NA
5-HT$_7$	506	3547	>1E4	>1E4	209	87.5	3.9	425	968.7	19.8	3423	1231	283.9	5769	1591	>1E4	6.6	NA
D$_1$	>1E4	>1E4	>1E4	>1E4	>1E4	271	79.5	>1E4	>1E4	>1E4	>1E4	>1E4	>1E4	9687.5	>1E4	>1E4	177	>1E4
D$_2$	>1E4	>1E4	>1E4	>1E4	3371	>1E4	3562	3339	2795	>1E4	>1E4	>1E4	9249	>1E4	>1E4	>1E4	110.1	>1E4
D$_3$	808	>1E4	>1E4	>1E4	7116	>1E4	497	1345	1835	2470	3321	>1E4	1361	>1E4	>1E4	>1E4	27	>1E4
D$_4$	>1E4	>1E4	>1E4	>1E4	>1E4	>1E4	3120	>1E4	>1E4	6331	>1E4	>1E4	2014	>1E4	>1E4	>1E4	158.4	>1E4
D$_5$	>1E4	>1E4	>1E4	>1E4	>1E4	>1E4	>1E4	>1E4	>1E4	>1E4	>1E4	>1E4	>1E4	>1E4	>1E4	>1E4	344.4	NA
α_{1A}	>1E4	>1E4	>1E4	>1E4	>1E4	1745	>1E4	>1E4	>1E4	>1E4	>1E4	>1E4	880.8	>1E4	3219	>1E4	1127.7	NA
α_{1B}	>1E4	>1E4	>1E4	>1E4	>1E4	973	>1E4	>1E4	>1E4	>1E4	>1E4	>1E4	443.4	>1E4	>1E4	>1E4	8677	NA
α_{2A}	4266	1655	2532	8930	308	1561	1890	490	729.7	175	>1E4	>1E4	457.6	73.7	579.9	>1E4	45.8	NA
α_{2B}	1527	690	1785	>1E4	664	257	2640	305	981.8	1693	2870	5293	339.3	340	873.7	>1E4	NA	NA
α_{2C}	593	229	1346	745.3	103	258	508	90	165.9	637	2523	2865	514.4	600.6	921.3	>1E4	NA	NA
β_1	2425	>1E4	>1E4	>1E4	>1E4	>1E4	>1E4	>1E4	9202	>1E4	>1E4	>1E4	>1E4	590.9	>1E4	>1E4	1600.7	>1E4
β_2	302	>1E4	>1E4	>1E4	>1E4	>1E4	>1E4	>1E4	1184	>1E4	>1E4	>1E4	>1E4	138.6	48.9	>1E4	3460.9	NA
SERT	8538	>1E4	>1E4	>1E4	>1E4	3742	2032	>1E4	>1E4	6409	1258	2531	156.7	684.7	>1E4	>1E4	>1E4	548.7
DAT	>1E4	>1E4	>1E4	>1E4	>1E4	>1E4	>1E4	>1E4	>1E4	>1E4	>1E4	>1E4	1500	>1E4	>1E4	>1E4	>1E4	1980
NET	>1E4	>1E4	>1E4	>1E4	>1E4	>1E4	2859	>1E4	>1E4	>1E4	>1E4	>1E4	>1E4	>1E4	>1E4	>1E4	>1E4	NA
I$_1$	1596	>1E4	219.7	2677	2155	650	2498	>1E4	2080	879.4	356	759.5	340	>1E4	>1E4	NA	NA	NA
σ_1	2193	NA	NA	>1E4	>1E4	5209	>1E4	>1E4	3870	>1E4	1798	9443	396.8	8565	>1E4	NA	NA	5839
σ_2	>1E4	NA	NA	>1E4	>1E4	>1E4	>1E4	>1E4	>1E4	917.9	2702	3002	2917	9172	>1E4	NA	NA	206
DOR	>1E4	NA	NA	>1E4	>1E4	NA	NA	>1E4	>1E4	>1E4	>1E4	>1E4	>1E4	>1E4	>1E4	NA	>1E4	>1E4
KOR	>1E4	>1E4	>1E4	>1E4	>1E4	>1E4	>1E4	>1E4	>1E4	>1E4	>1E4	>1E4	>1E4	>1E4	>1E4	NA	>1E4	2717
MOR	>1E4	NA	>1E4	>1E4	>1E4	NA	NA	>1E4	>1E4	>1E4	>1E4	>1E4	>1E4	>1E4	>1E4	NA	>1E4	4362
M$_1$	>1E4	NA	>1E4	>1E4	>1E4	>1E4	>1E4	>1E4	>1E4	>1E4	>1E4	>1E4	>1E4	2720	>1E4	NA	NA	>1E4
M$_2$	>1E4	>1E4	>1E4	>1E4	>1E4	>1E4	>1E4	>1E4	>1E4	>1E4	>1E4	>1E4	>1E4	1989	>1E4	>1E4	NA	>1E4
M$_3$	1151	NA	1851	>1E4	821	>1E4	>1E4	2556.3	692.2	>1E4	>1E4	>1E4	>1E4	1428	NA	NA	NA	>1E4
M$_4$	>1E4	NA	8245	>1E4	>1E4	>1E4	>1E4	>1E4	>1E4	>1E4	>1E4	>1E4	>1E4	577.7	NA	NA	NA	NA
M$_5$	>1E4	>1E4	6330	>1E4	>1E4	>1E4	>1E4	1725	1502	>1E4	>1E4	>1E4	>1E4	2208	>1E4	>1E4	NA	NA
H$_1$	9120	NA	>1E4	>1E4	>1E4	NA	NA	>1E4	>1E4	4819	3583	>1E4	125.1	1757	>1E4	1407	1543.5	>1E4
H$_2$	>1E4	>1E4	>1E4	>1E4	>1E4	>1E4	>1E4	>1E4	>1E4	>1E4	>1E4	>1E4	>1E4	>1E4	>1E4	>1E4	NA	NA
CB$_1$	>1E4	>1E4	>1E4	>1E4	>1E4	>1E4	>1E4	>1E4	>1E4	NA	>1E4	>1E4	>1E4	>1E4	>1E4	NA	NA	NA
CB$_2$	>1E4	>1E4	NA	>1E4	>1E4	>1E4	>1E4	>1E4	>1E4	>1E4	>1E4	>1E4	>1E4	>1E4	>1E4	NA	NA	NA
Ca+	>1E4	NA	1198	>1E4	NA	>1E4	>1E4	>1E4	>1E4	>1E4	>1E4	>1E4	>1E4	NA	NA	NA	NA	NA
NMDA	>1E4	NA	>1E4	>1E4	>1E4	NA	NA	>1E4	>1E4	>1E4	>1E4	>1E4	>1E4	>1E4	>1E4	NA	>1E4	2001

Table S1: Raw binding affinity data (nm) for all compounds and binding sites. “NA“ indicates missing data. As described in the methods section, binding affinities were capped at 10.000 nM. Table adapted from Ray (2010).

Binding site	DOB	MDA	MDMA	Mescaline	2C-B	DMT	5-MeO-DMT	2C-E	2C-T-2	5-MeO-MIPT	DIPT	5-MeO-DIPT	DPT	DOI	DOM	TMA-2	LSD	Ibogaine
5-HT_{1A}	3.4	3.6	4.0	3.3	2.4	4.0	0.3	2.5	2.6	1.1	2.1	2.1	1.5	3.3	3.6	4.0	0.9	4.0
5-HT_{1B}	3.0	4.0	4.0	4.0	2.0	4.0	1.9	2.4	2.9	2.5	4.0	3.7	2.9	4.0	4.0	4.0	0.6	4.0
5-HT_{1D}	2.8	4.0	4.0	4.0	1.4	2.0	0.8	1.9	1.9	1.4	3.6	3.2	2.8	2.7	2.3	4.0	0.9	4.0
5-HT_{1E}	3.2	4.0	4.0	3.7	2.1	2.7	2.6	2.8	2.6	3.5	4.0	4.0	3.4	3.0	3.5	4.0	2.0	NA
5-HT_{2A}	1.4	4.0	4.0	4.0	1.4	3.4	3.3	1.6	1.6	2.7	4.0	4.0	3.4	2.2	2.7	2.8	1.1	4.2
5-HT_{2B}	0.6	2.0	2.7	2.9	1.1	2.0	3.6	1.4	0.8	1.8	2.6	2.2	1.6	2.5	1.1	2.2	1.5	NA
5-HT_{2C}	1.6	3.8	4.0	4.0	2.0	2.5	2.7	2.0	1.7	3.3	4.0	4.0	3.2	1.7	3.6	3.6	1.5	4.0
5-HT_{5A}	3.7	4.0	4.0	4.0	4.0	2.8	2.4	4.0	4.0	3.0	4.0	4.0	3.6	4.0	4.0	4.0	1.0	NA
5-HT_6	3.7	4.0	4.0	4.0	2.5	2.7	1.6	3.5	3.1	2.1	4.0	4.0	3.7	3.3	3.9	4.0	0.8	NA
5-HT_7	2.7	3.5	4.0	4.0	2.3	1.9	0.6	2.6	3.0	1.3	3.5	3.1	2.5	3.8	3.2	4.0	0.8	NA
D_1	4.0	4.0	4.0	4.0	4.0	2.4	1.9	4.0	4.0	4.0	4.0	4.0	4.0	4.0	4.0	4.0	2.2	4.0
D_2	4.0	4.0	4.0	4.0	3.5	4.0	3.6	3.5	3.4	4.0	4.0	4.0	4.0	4.0	4.0	4.0	2.0	4.0
D_3	2.9	4.0	4.0	4.0	3.9	4.0	2.7	3.1	3.3	3.4	3.5	4.0	3.1	4.0	4.0	4.0	1.4	4.8
D_4	4.0	4.0	4.0	4.0	4.0	4.0	3.5	4.0	4.0	3.8	4.0	4.0	3.3	4.0	4.0	4.0	2.2	4.0
D_5	4.0	4.0	4.0	4.0	4.0	4.0	4.0	4.0	4.0	4.0	4.0	4.0	4.0	4.0	4.0	4.0	2.5	NA
α_{1A}	4.0	4.0	4.0	4.0	4.0	3.2	4.0	4.0	4.0	4.0	4.0	4.0	2.9	4.0	3.5	4.0	3.1	NA
α_{1B}	4.0	4.0	4.0	4.0	4.0	3.0	4.0	4.0	4.0	4.0	4.0	4.0	2.6	4.0	4.0	4.0	3.9	NA
α_{2A}	3.6	3.2	3.4	4.0	2.5	3.2	3.3	2.7	2.9	2.2	4.0	4.0	2.7	1.9	2.8	4.0	1.7	NA
α_{2B}	3.2	2.8	3.3	4.0	2.8	2.4	3.4	2.5	3.0	3.2	3.5	3.7	2.5	2.5	2.9	4.0	NA	NA
α_{2C}	2.8	2.4	3.1	2.9	2.0	2.4	2.7	2.0	2.2	2.8	3.4	3.5	2.7	2.8	3.0	4.0	NA	NA
β_1	3.4	4.0	4.0	4.0	4.0	4.0	4.0	4.0	4.0	4.0	4.0	4.0	4.0	2.8	4.0	4.0	3.2	4.0
β_2	2.5	4.0	4.0	4.0	4.0	4.0	4.0	4.0	3.1	4.0	4.0	4.0	4.0	2.1	1.7	4.0	3.5	NA
SERT	3.9	4.0	4.0	4.0	4.0	3.6	3.3	4.0	4.0	3.8	3.1	3.4	2.2	2.8	4.0	4.0	4.0	2.7
DAT	4.0	4.0	4.0	4.0	4.0	4.0	4.0	4.0	4.0	4.0	4.0	4.0	3.2	4.0	4.0	4.0	4.0	3.3
NET	4.0	4.0	4.0	4.0	4.0	4.0	3.5	4.0	4.0	4.0	4.0	4.0	4.0	4.0	4.0	4.0	4.0	NA
I_1	3.2	4.0	2.3	3.4	3.3	2.8	3.4	4.0	3.3	2.9	2.6	2.9	2.5	4.0	4.0	NA	NA	NA
σ_1	3.3	NA	NA	4.0	4.0	3.7	4.0	4.0	3.6	4.0	3.3	4.0	2.6	3.9	4.0	NA	NA	3.8
σ_2	4.0	NA	NA	4.0	4.0	4.0	4.0	4.0	4.0	3.0	3.4	3.5	3.5	4.0	4.0	NA	NA	2.3
DOR	4.0	NA	NA	4.0	4.0	NA	NA	4.0	4.0	4.0	4.0	4.0	4.0	4.0	4.0	NA	4.0	4.0
KOR	4.0	4.0	4.0	4.0	4.0	4.0	4.0	4.0	4.0	4.0	4.0	4.0	4.0	4.0	4.0	NA	4.0	3.4
MOR	4.0	NA	4.0	4.0	4.0	NA	NA	4.0	4.0	4.0	4.0	4.0	4.0	4.0	4.0	NA	4.0	3.6
M_1	4.0	NA	4.0	4.0	4.0	4.0	4.0	4.0	4.0	4.0	4.0	4.0	4.0	3.4	4.0	NA	NA	4.4
M_2	4.0	4.0	4.0	4.0	4.0	4.0	4.0	4.0	4.0	4.0	4.0	4.0	4.0	3.3	4.0	4.0	NA	4.6
M_3	3.1	NA	3.3	4.0	2.9	4.0	4.0	3.4	2.8	4.0	4.0	4.0	4.0	3.2	NA	NA	NA	4.1
M_4	4.0	NA	3.9	4.0	4.0	4.0	4.0	4.0	4.0	4.0	4.0	4.0	4.0	2.8	NA	NA	NA	NA
M_5	4.0	4.0	3.8	4.0	4.0	4.0	4.0	3.2	3.2	4.0	4.0	4.0	4.0	3.3	4.0	4.0	NA	NA
H_1	4.0	NA	4.0	4.0	4.0	NA	NA	4.0	4.0	3.7	3.6	4.0	2.1	3.2	4.0	3.1	3.2	4.0
H_2	4.0	4.0	4.0	4.0	4.0	4.0	4.0	4.0	4.0	4.0	4.0	4.0	4.0	4.0	4.0	4.0	NA	NA
CB_1	4.0	4.0	4.0	4.0	4.0	4.0	4.0	4.0	4.0	NA	4.0	4.0	4.0	4.0	4.0	NA	NA	NA
CB_2	4.0	4.0	NA	4.0	4.0	4.0	4.0	4.0	4.0	4.0	4.0	4.0	4.0	4.0	4.0	NA	NA	NA
Ca+	4.0	NA	3.1	4.0	NA	4.0	4.0	4.0	4.0	4.0	4.0	4.0	4.0	NA	NA	NA	NA	NA
NMDA	4.0	NA	4.0	4.0	4.0	NA	NA	4.0	4.0	4.0	4.0	4.0	4.0	4.0	4.0	NA	4.0	3.3

Table S2: Normalized binding affinity data for all compounds and binding sites. Values represent $pK_i = -\log_{10}(K_i)$ without inclusion of the minus sign, since it is present for all values. “NA“ indicates missing data. As described in the Methods section, normalized binding affinities were capped at -4. Table adapted from Ray (2010).
