# Supplementary material for: The Varieties of the Psychedelic Experience: A Preliminary Study of the Association Between the Reported Subjective Effects and the Binding Affinity Profiles of Substituted Phenethylamines and Tryptamines
Source: Front Integr Neurosci. 2018 Nov 8;12:54. doi: 10.3389/fnint.2018.00054 (PMC6235949; doi:10.3389/fnint.2018.00054)
Supplement: Supplementary file 2 [file Data_Sheet_2.PDF]

Supplementary figure: robustness against the normalization of Ki values procedure

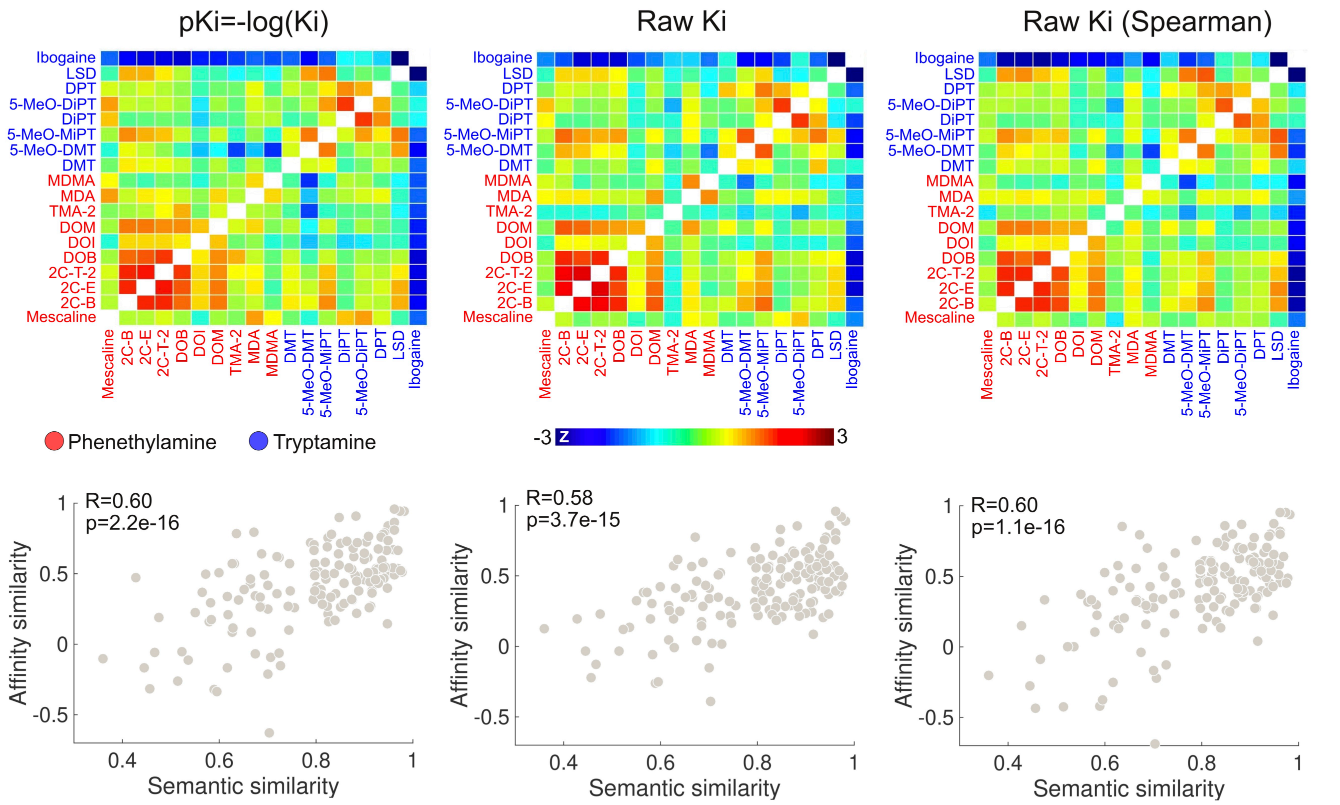

Figure S1: The upper panel (left) shows the affinity similarity matrices obtained from the pKi values (as defined in the manuscript text, thus concordant with the resuls shown in **Figure 4** of the manuscript), from the raw (i.e. unnormalized) Ki values (middle), and from the raw Ki values using Spearman's correlation instead of Pearson's correlation to compare the affinity profiles (right). The bottom panel presents scatter plots for the affinity similarities (computed using each of the three normalization procedures) and the semantic similarities. In all cases, we observed R ≈0.6 with a highly significant p-value.
